# Attribute-Aware Recommender System Based on Collaborative Filtering: Survey and Classification

**DOI:** 10.3389/fdata.2019.00049

**Published:** 2020-01-15

**Authors:** Wen-Hao Chen, Chin-Chi Hsu, Yi-An Lai, Vincent Liu, Mi-Yen Yeh, Shou-De Lin

**Affiliations:** ^1^Department of Computer Science and Information Engineering, National Taiwan University, Taipei, Taiwan; ^2^Institute of Information Science, Academia Sinica, Taipei, Taiwan

**Keywords:** recommender system, matrix factorization, collaborative filtering, attribute, survey

## Abstract

Attribute-aware CF models aim at rating prediction given not only the historical rating given by users to items but also the information associated with users (e.g., age), items (e.g., price), and ratings (e.g., rating time). This paper surveys work in the past decade to develop attribute-aware CF systems and finds that they can be classified into four different categories mathematically. We provide readers not only with a high-level mathematical interpretation of the existing work in this area but also with mathematical insight into each category of models. Finally, we provide in-depth experiment results comparing the effectiveness of the major models in each category.

## 1. Introduction

Collaborative filtering is arguably the most effective method for building a recommender system. It assumes that a user's preferences regarding items can be inferred collaboratively from other users' preferences. In practice, users' past records regarding items, such as explicit ratings or implicit feedback (e.g., binary access records), are typically used to infer similarity of taste among users for the purposes of recommendation. In the past decade, *matrix factorization* (MF) has become a widely adopted method of collaborative filtering. Specifically, MF learns a latent representation vector for a user and an item and computes their inner products as the predicted rating. The learned latent user/item factors are supposed to embed specific information about the user/item. That is, two users with similar latent representations will have similar tastes regarding items with similar latent vectors.

**Figure 1:**
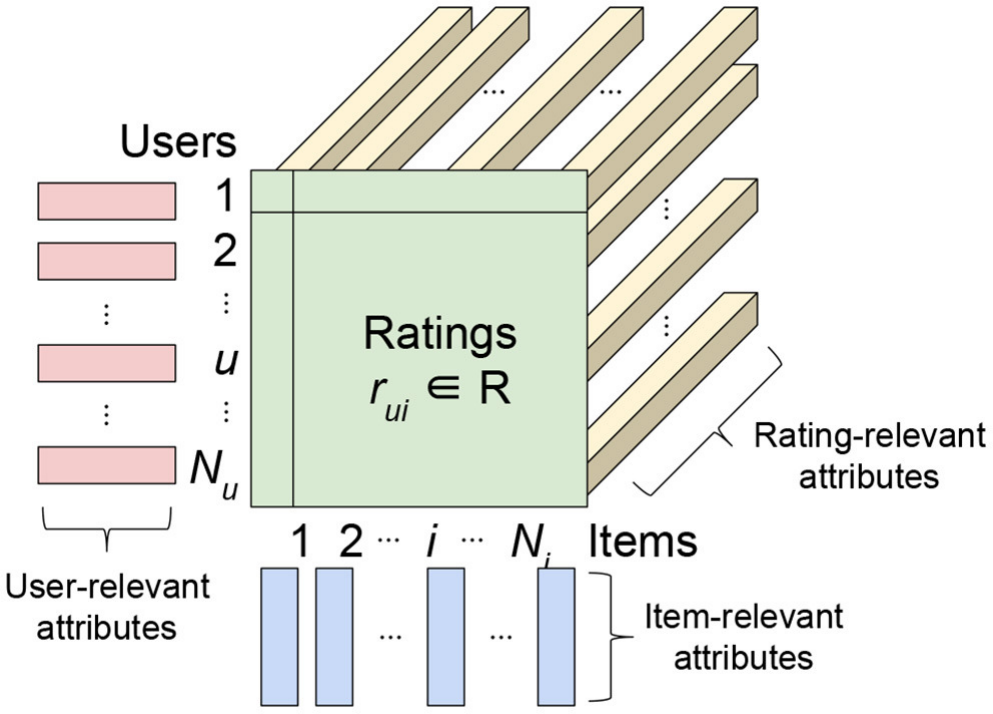
Interpretation of inputs, including ratings and attributes, in attribute-aware collaborative filtering-based recommender systems.

In the big data era, classical MF using only ratings suffers a serious drawback, as such a method is unable to exploit other accessible information such as the attributes of users/items/ratings. For instance, data could contain the location and time that a user rated an item. These rating-relevant attributes, or *contexts*, could be useful in determining the scale of user liking for an item. The *side information* or attributes relevant to users or items (e.g., the demographic information of users or the item genre) can also reveal useful information. Such side information is particularly useful for situations where the ratings of a user or an item are sparse, which is known as the *cold-start* problem for recommender systems. Therefore, researchers have formulated *attribute-aware recommender systems* (see Figure 1) aiming at leveraging not only the rating information but also the attributes associated with ratings/users/items to improve the quality of recommendation.

Researchers have proposed different methods to extend existing collaborative filtering models in recent years, such as factorization machines, probabilistic graphical models, kernel tricks, and models based on deep neural networks. We notice that those papers can also be categorized based on the type of attributes incorporated into the models. A class of recommender systems considers relevant side information, such as age, gender, the occupation of users, or the expiration and price of items when predicting ratings (e.g., Adams et al., 2010; Porteous et al., 2010; Fang and Si, 2011; Ning and Karypis, 2012; Zhou et al., 2012; Park et al., 2013; Xu et al., 2013; Kim and Choi, 2014; Lu et al., 2016; Zhao et al., 2016; Feipeng Zhao, 2017; Guo, 2017; Yu et al., 2017; Zhou T. et al., 2017). On the other hand, *context-aware recommender systems* (e.g., Shin et al., 2009; Karatzoglou et al., 2010; Li et al., 2010b; Baltrunas et al., 2011; Rendle et al., 2011; Hidasi and Tikk, 2012, 2016; Shi et al., 2012a, 2014a; Liu and Aberer, 2013; Chen et al., 2014; Nguyen et al., 2014; Hidasi, 2015; Liu and Wu, 2015) enhance themselves by considering the attributes appended to each rating (e.g., rating time, rating location). Other terms may be used to indicate attributes interchangeably such as *metadata* (Kula, 2015), *features* (Chen et al., 2012), *taxonomy* (Koenigstein et al., 2011), *entities* (Yu et al., 2014), *demographic data* (Safoury and Salah, 2013), *categories* (Chen et al., 2016), *contexture information* (Weng et al., 2009), etc. The above setups all share the same mathematical representation; thus, technically, we do not distinguish them in this paper. That is, we regard whichever information is associated with a user/item/rating as user/item/rating attributes, regardless of its nature. Therefore, a CF model that takes advantage of ratings as well as associated attributes is called an *attribute-aware recommender* in this paper.

Note that the attribute-aware recommender systems discussed in this paper are not equivalent to hybrid recommender systems. The former treat additional information as attributes, while the latter emphasize the combination of collaborative filtering-based methods and content-based methods. To be more precise, this review covers only works that assume unstructured and independent attributes, either in binary or numerical format, for each user, item, or rating. The reviewed models do not have prior knowledge of the dependency between attributes, such as the adjacent terms in a document or user relationships in a social network.

This review covers more than one hundred papers in this area in the past decade. We find that the majority of the works propose an extension of matrix factorization to incorporate attribute information in collaborative filtering. The main contribution of this paper is to not only provide a comprehensive review, but also provide a means to classify these works into four categories: (I) *discriminative matrix factorization*, (II) *generative matrix factorization*, (III) *generalized factorization*, and (IV) *heterogeneous graphs*. For each category, we provide the probabilistic interpretation of the models. The major distinction of these four categories lies in their representation of the interactions between users, items, and attributes. Discriminative matrix factorization models extend the traditional MF by treating the attributes as prior knowledge to learn the latent representation of users or items. Generative matrix factorization further considers the distributions of attributes and learns such, together with the rating distributions. Generalized factorization models view the user/item identity simply as a kind of attribute, and various models have been designed for determining the low-dimensional representation vectors for rating prediction. The last category of models proposes to represent the users, items, and attributes using a heterogeneous graph, where a recommendation task can be cast into a link-prediction task on the heterogeneous graph. In the following sections, we will elaborate on general mathematical explanations of the four types of model designs and discuss the similarities/differences among the models.

There have been four prior reviews (Adomavicius and Tuzhilin, 2011; Verbert et al., 2012; Bobadilla et al., 2013; Shi et al., 2014b) introducing attribute-aware recommender systems. We claim three major differences between our work and the existing papers. First, previous review papers mainly focused on grouping different types of attributes and discussing the distinctions of memory-based collaborative filtering and model-based collaborative filtering. In contrast, we are the first that have aimed at classifying the existing works based on the methodology proposed instead of the type of data used. We further provide mathematical connections for different types of models so that the readers can better understand the spirit of the design of different models as well as their technical differences. Second, we are the first to provide thorough experiment results (seven different models on eight benchmark datasets) to compare different types of attribute-award recommendation systems. Note that Bobadilla et al. (2013) is the only previous review work with experimental results. However, for that study, experiments were performed to compare different similarity measures in collaborative filtering algorithms instead of directly verifying the effectiveness of different attribute-aware recommender systems. Finally, we cover the latest work on attribute-aware recommender systems. We note that the existing review papers do not include *forty* papers after 2015. In recent years, several deep neural network-based solutions (Zhang et al., 2017) have achieved state-of-the-art performance for this task.

Table 1 shows comparisons between our work and previous reviews.

**Table 1:** Differences between previous works and our work.

**Difference**	**Previous works (Adomavicius and Tuzhilin, 2011; Verbert et al., 2012; Bobadilla et al., 2013; Shi et al., 2014b)**	**Our work**
Attribute discussions	Categories and definitions of diversified attributes	Mathematical formulations of the most general attribute vectors
Model introduction	High-level summary of text descriptions	Mathematical interpretation of model design criteria
Comparative experiments	For memory-based models in Bobadilla et al. (2013); no experiments in others	For seven model-based models on seven benchmark datasets

We will introduce the basic concepts behind recommender systems in section 2, followed by formal analyses of attribute-aware recommender systems in sections 3 and 4. A series of experiments detailed in section 5 were conducted to compare the accuracy and parameter sensitivity of six widely adopted models. Finally, section 6 concludes this review and identifies work to be done in the future.

## 2. Preliminaries

### 2.1. Problem Definition of Recommender Systems

*Recommender systems* act as skilled agents to assist *users* to conquer information overload while making selection decisions over *items* by providing customized recommendations. *Users* and *items* are general phrases denoting, respectively, entities actively browsing and making choices and entities being selected, such as goods and services.

Formally, recommender systems leverage one or more of the following three information sources to discover user preferences and generate recommendations: *user-item interactions, side information*, and *contexts*. *User-item interactions*, or *ratings*, are collected explicitly by prompting users to provide numerical feedback on items and are acquired implicitly by tracking user behaviors such as clicks, browsing time, or purchase history. The data are commonly represented as a matrix that encodes the preferences of users and is naturally sparse, since users normally interact with a limited fraction of items. *Side information* is rich information attached to an individual user or item that depicts user characteristics such as education and job or item properties such as description and product categories. Side information can take diverse structures with rich meaning, ranging over numerical status, texts, and images to videos, locations, and networks. On the other hand, *context* refers to all the information collected when a user interacts with an item, such as timestamps, locations, or textual reviews. This contextual information usually serves as an additional information source appended to the user-item interaction matrix.

The goal of recommender systems is to disclose unknown user preferences over items that users never interact with and recommend the most preferred items to them. In practice, recommender systems learn to generate recommendations based on three types of approaches: *pointwise, pairwise*, and *listwise*. The *pointwise* approach is the most common approach and demands recommendation systems to provide accurate numerical predictions on observed ratings. Items that a user never interacts with are then sorted by their rating predictions, and a number of items with the highest ratings are recommended to the user. On the other hand, a *pairwise* approach seeks to preserve the ordering of any pair of items based on ratings, while in the *listwise* approach, recommender systems aim to preserve the relative order of all rated items as a list for each user. The pairwise approach and listwise approach are together considered *item rankings* that only require recommender systems to output the ordering of items but not ratings for individual items.

In most of the works covered in this paper, the task of attribute-aware recommendation is defined as *predicting unknown ratings*, that is: given *N*_*u*_ users, *N*_*i*_ items, a *user-item rating* matrix $\text{R}\in {\mathbb{R}}^{N_{u}\times N_{i}}$ with only a small portion *N*_*r*_ ratings observed (i.e., there are a total of *N*_*u*_ × *N*_*i*_ − *N*_*r*_ missing ratings), *side information of users* $\text{X}\in {\mathbb{R}}^{K_{X}\times N_{u}}$ (assuming each user has *K*_*X*_ attributes), *side information of items* $\text{Y}\in {\mathbb{R}}^{K_{Y}\times N_{i}}$, and *contexts* $\text{Z}\in {\mathbb{R}}^{K_{Z}\times N_{r}}$, the goal is to build a model that is capable of predicting each of the unknown ratings in ***R***.

Then, given any specific user, a recommender system can make recommendations based on the predicted ratings. Normally, items with higher ratings are recommended first. Note that the dimension *K*_*X*_, *K*_*Y*_ of side information attribute matrix ***X***, ***Y*** might be zero, denoting that there is no side information about users or items. Likewise, if there is no contextual information about user-item interactions, *K*_*Z*_ will be zero.

The core techniques or algorithms for realizing recommender systems are generally classified into three categories: *content-based filtering, collaborative filtering*, and *hybrid filtering* (Bobadilla et al., 2013; Shi et al., 2014b; Isinkaye et al., 2015). *Content-based filtering* generates recommendations based on properties of items and user-item interactions. Content-based techniques exploit domain knowledge and seek to transform item properties in raw attribute structures such as texts, images, or locations into numerical item profiles. Each item is represented as a vector, and the matrix of side information of items ***Y*** is constructed. A representation of each user is then created by aggregating profiles of items that this user interacted with, and a similarity measure is leveraged to retrieve a number of the most similar items as recommendations. Note that content-based filtering does not require information from any other user to make recommendations. *Collaborative filtering* strives to identify a group of users with similar preferences based on past user-item interactions and recommends items preferred by these users. Since discovering users with common preferences is generally based on user-item ratings ***R***, collaborative filtering becomes the first choice when item properties are inadequate in describing their content, such as movies or songs. *Hybrid filtering* is the extension or combination of content-based and collaborative filtering. Examples of this are building an ensemble of the two techniques, using the item rating history of collaborative filtering as part of the item profiles for content-based filtering or extending collaborative filtering to incorporate user characteristics ***X*** or item properties ***Y***. This review focuses on *attribute-aware recommender systems* that shed light not only on user-item interactions ***R*** but also on the side information of users or items ***X***, ***Y*** and contexts ***Z***, which is a subset of hybrid filtering.

### 2.2. Collaborative Filtering and Matrix Factorization

Collaborative filtering (CF) has become the prevailing technique for realizing recommender systems in recent years (Adomavicius and Tuzhilin, 2005, 2011; Shi et al., 2014b; Isinkaye et al., 2015). It assumes that the preferences users exhibit for items they have interacted with can be generalized and used to infer their preferences regarding items they have never interacted with by leveraging the records of other users with similar preferences. This section briefly introduces conventional CF techniques, which assume the availability of only user-item interactions or the rating matrix ***R***. In practice, they are commonly categorized into *memory-based* CF and *model-based* CF (Adomavicius and Tuzhilin, 2005; Shi et al., 2014b; Isinkaye et al., 2015).

*Memory-based* CF directly exploits rows or columns in the rating matrix ***R*** as representations of users or items and identifies a group of similar users or items using a pre-defined similarity measure. Commonly used similarity metrics include the Pearson correlation, the Jaccard similarity coefficient, the cosine similarity, or their variants. Memory-based CF techniques can be divided into *user-based* and *item-based* approaches, which identify either a group of similar users or similar items, respectively. For user-based approaches, *K* nearest neighbors—or the *K* most similar users—are extracted, and their preferences or ratings regarding a target item are aggregated into a rating prediction using similarities between users as weights. The rating prediction for user *u* on item *i*, ${\hat{r}}_{ui}$, can be formulated as:

(1) $$\begin{array}{l} {\hat{r}}_{ui}=\frac{1}{Z}\underset{v\in U_{u}}{\sum }\text{sim}\left(u,v\right)r_{vi}, \end{array}$$

where function sim(·) is a similarity measure, *Z* is the normalization constant, and *U*_*u*_ is the set of similar users to user *u* (Shi et al., 2014b). Rating predictions of item-based approaches can be formulated in a similar way. The calculated pairwise similarities between users or items act as the *memory* of the recommender system since they can be saved for generating later recommendations.

*Model-based* CF, on the other hand, takes the rating matrix ***R*** to train a predictive model with a set of parameters ***θ*** to make recommendations (Adomavicius and Tuzhilin, 2005; Shi et al., 2014b). Predictive models can be formulated as a function that outputs ratings for *rating predictions* or numerical preference scores for *item ranking* given a user-item pair (*u, i*):

(2) $$\begin{array}{l} {\hat{r}}_{ui}=f_{\theta }\left(u,i\right). \end{array}$$

Model-based CF then ranks and selects the *K* items with the highest ratings or scores *r*_*ui*_ as recommendations. Common core algorithms for model-based CF involve Bayesian classifiers, clustering techniques, graph-based approaches, genetic algorithms, and dimension-reduction methods such as Singular Value Decomposition (SVD) (Adomavicius and Tuzhilin, 2005, 2011; Bobadilla et al., 2013; Shi et al., 2014b; Isinkaye et al., 2015). Over the last decade, a class of *latent factor models*, called *matrix factorization*, has been popularized and is commonly adopted as the basis of advanced techniques because of its success in the development of algorithms for the Netflix competition (Koren et al., 2009; Koren and Bell, 2011). In general, latent factor models aim to learn a low-dimensional representation, or latent factor, for each entity and combine the latent factors of different entities using specific methods such as inner product, bilinear map, or neural networks to make predictions. As one of these latent factor models, matrix factorization for recommender systems characterizes each user and item by a low-dimensional vector and predicts ratings based on inner product.

*Matrix factorization* (MF) (Paterek, 2007; Koren et al., 2009; Koren and Bell, 2011; Shi et al., 2014b), in the basic form, represents each user *u* as a parameter vector ${\text{w}}_{u}\in {\mathbb{R}}^{K}$ and each item *i* as ${\text{h}}_{i}\in {\mathbb{R}}^{K}$, where *K* is the dimension of latent factors. The prediction of user *u*'s rating or preference regarding item *i*, denoted as ${\hat{r}}_{ui}$, can be computed using the inner product:

(3) $$\begin{array}{l} {\hat{r}}_{ui}=w_{u}^{⊤}h_{i}, \end{array}$$

which captures the interaction between them. MF seeks to generate rating predictions that are as close as possible to those recorded ratings. In matrix form, it can be written as finding ***W***, ***H*** such that ***R*** ≈ ***W***^⊤^***H*** where $\text{R}\in {\mathbb{R}}^{N_{u}\times N_{i}}$. MF is essentially learning a low-rank approximation of the rating matrix, since the dimension of representations *K* is usually much smaller than the number of users *N*_*u*_ and items *N*_*i*_. To learn the latent factors of users and items, the system tries to find ***W***, ***H*** that minimizes the regularized square error on the set of entries of known ratings in ***R*** [denoted as δ(***R***)]:

(4) $$\begin{array}{l} W^{*},\;H^{*}=\underset{W,H}{\mathrm{argmin}}\underset{\left(u,i\right)\in \delta \left(R\right)}{\sum }\frac{1}{2}{\left(r_{ui}-w_{u}^{⊤}h_{i}\right)}^{2}+\frac{\lambda_{W}}{2}\overset{N_{u}}{\underset{u=1}{\sum }}||w_{u}{||}_{2}^{2}\\ \;+\frac{\lambda_{H}}{2}\overset{N_{i}}{\underset{i=1}{\sum }}||h_{i}{||}_{2}^{2}, \end{array}$$

where λ_*W*_ and λ_*H*_ are regularization parameters. MF tends to cluster users or items with similar rating configurations into groups in the latent factor space, which implies that similar users or items will be close to each other. Furthermore, MF assumes that the rank of rating matrix ***R*** or the dimension of the vector space generated by the rating configuration of users is far smaller than the number of users *N*_*u*_. This implies that each user's rating configuration can be obtained by a linear combination of ratings from a group of other users since they are all generated by *K* principal vectors. Thus, MF is in the spirit of collaborative filtering, which is to infer a user's unknown ratings by the ratings of several other users.

*Biased matrix factorization* (Paterek, 2007; Koren et al., 2009; Koren and Bell, 2011), an improvement of MF, models the characteristics of each user and each item and the global tendency that are independent of user-item interactions. The obvious drawback of MF is that only user-item interactions ${\text{w}}_{u}^{⊤}{\text{h}}_{i}$ are considered in rating predictions. However, ratings usually contain universal shifts or exhibit systematic tendencies with respect to users and items. For instance, there might be a group of users inclined to give significantly higher ratings than others or a group of items widely considered to be of high-quality and that receive higher ratings. Besides, it is common that all ratings are non-negative, which implies that the overall average might not be close to zero and causes a difficulty for training small-value-initialized representations. Due to the issues mentioned above, biased MF augments MF rating predictions with linear biases that account for user-related, item-related, and global effects. The rating prediction is extended as follows:

(5) $$\begin{array}{l} {\hat{r}}_{ui}=\mu +c_{u}+d_{i}+w_{u}^{⊤}h_{i}, \end{array}$$

where μ, *c*_*i*_, *d*_*j*_ are global bias, bias of user *i*, and bias of item *j*, respectively. Biased MF then finds the optimal ***W***, ***H***, ***c***, ***d***, μ that minimizes the regularized square error as follows:

(6) $$\begin{array}{l} W^{*},H^{*},c^{*},d^{*},\mu^{*}\\ =\underset{W,H,c,d,\mu }{\text{argmin}}\sum_{\left(u,i)\in \delta (R\right)}{\left(r_{ui}-\mu -c_{u}-d_{i}-w_{u}^{⊤}h_{i}\right)}^{2}+\text{λ}(||W{||}_{F}^{2}\\ \;+||H{||}_{F}^{2}+||c{||}_{2}^{2}+||d{||}_{2}^{2}), \end{array}$$

where $||\text{W}{||}_{F}^{2}=\overset{N_{u}}{\underset{u=1}{\sum }}||{\text{w}}_{u}{||}_{2}^{2}$ denotes the squared Frobenius norm. The regularization parameter λ is tuned by cross-validation.

**Figure 2:**
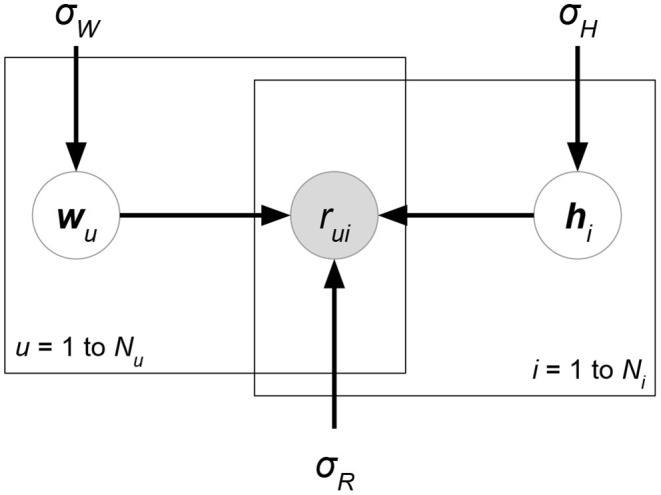
PMF.

**Figure 3:**
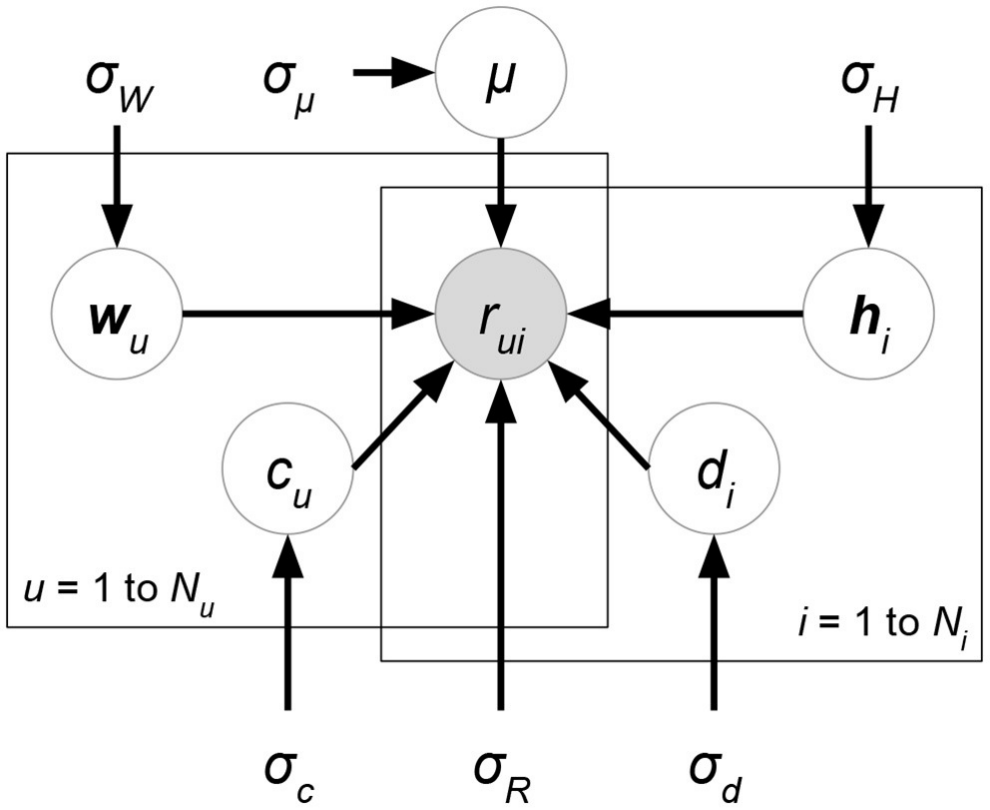
Biased PMF.

*Probabilistic matrix factorization* (PMF, Figure 2 and Biased PMF, Figure 3) (Salakhutdinov and Mnih, 2007, 2008) is a probabilistic linear model with observed Gaussian noise and can be viewed as a probabilistic extension of MF. PMF adopts the assumption that users and items are independent and represents each user or each item with a zero-mean spherical multivariate Gaussian distribution as follows:

(7) $$\begin{array}{l} p\left(W|\sigma_{W}^{2}\right)=\prod_{u=1}^{N_{u}}N\left(w_{u}|0,\sigma_{W}^{2}I\right),p\left(H|\sigma_{H}^{2}\right)\\ \;=\overset{N_{i}}{\underset{i=1}{\prod }}N\left(h_{i}|0,\sigma_{H}^{2}I\right), \end{array}$$

where $\sigma_{W}^{2}$ and $\sigma_{H}^{2}$ are observed user-specific and item-specific noise. PMF then formulates the conditional probability over the observed ratings as

(8) $$\begin{array}{l} p\left(R|W,H,\sigma^{2}\right)=\underset{\left(i,j\right)\in \delta \left(R\right)}{\prod }N\left(r_{ui}|w_{u}^{⊤}h_{i},\sigma_{R}^{2}\right), \end{array}$$

where δ(***R***) is the set of known ratings and $N\left(x|\mu ,\sigma^{2}\right)$ denotes the Gaussian distribution with mean μ and variance σ^2^. Learning in PMF is conducted by maximum a posteriori (MAP) estimation, which is equivalent to maximizing the log of the posterior distribution of ***W***, ***H***:

(9) $$\begin{array}{l} \log p\left(W,H|R,\sigma_{R}^{2},\sigma_{W}^{2},\sigma_{H}^{2}\right)=\log p\left(R|W,H,\sigma_{R}^{2}\right)\\ \;+\log p\left(W|\sigma_{W}^{2}\right)+\log p\left(H|\sigma_{H}^{2}\right)+C\\ \;=-\frac{1}{2\sigma_{R}^{2}}\sum_{\left(u,i)\in \delta (R\right)}{\left(r_{ui}-w_{u}^{⊤}h_{i}\right)}^{2}-\frac{1}{2\sigma_{W}^{2}}\sum_{u=1}^{N_{u}}w_{u}^{⊤}w_{u}\\ \;-\frac{1}{2\sigma_{H}^{2}}\sum_{i=1}^{N_{i}}h_{i}^{⊤}h_{i}-\frac{1}{2}(|\delta (R)|\log\sigma_{R}^{2}+N_{u}K\log\sigma_{W}^{2}\\ \;+N_{i}K\log\sigma_{H}^{2})+C \end{array}$$

where *C* is a constant independent of all parameters and *K* is the dimension of user or item representations. With Gaussian noise $\sigma_{R}^{2},\sigma_{W}^{2},\sigma_{H}^{2}$ observed, maximizing the log-posterior is identical to minimizing the objective function with the form:

(10) $$\begin{array}{l} \underset{\left(u,i\right)\in \delta \left(R\right)}{\sum }\frac{1}{2}{\left(r_{ui}-w_{u}^{⊤}h_{i}\right)}^{2}+\frac{\lambda_{W}}{2}\overset{N_{u}}{\underset{u=1}{\sum }}||w_{u}{||}_{2}^{2}+\frac{\lambda_{H}}{2}\overset{N_{i}}{\underset{i=1}{\sum }}||h_{i}{||}_{2}^{2}, \end{array}$$

where $\lambda_{W}=\sigma_{R}^{2}/\sigma_{W}^{2},\lambda_{H}=\sigma_{R}^{2}/\sigma_{H}^{2}$. Note that (10) has exactly the same form as the regularized square error of MF, and gradient descent or its extensions can then be applied in training PMF.

Since collaborative filtering techniques only consider rating matrix ***R*** in making recommendations, they cannot discover the preferences of users or for items with scant user-item interactions. This problem is referred as the *cold-start issue*. In section 3, we will review recommendation systems that extend CF to incorporate contexts or rich side information regarding users and items to alleviate the cold-start problem.

## 3. Attribute-Aware Recommender Systems

### 3.1. Overview

Attribute-aware recommendation models are proposed to tackle the challenges of integrating additional information from user/item/rating. There are two strategies for designing attribute-aware collaborative filtering-based systems. One direction is to combine content-based recommendation models with CF models, which can directly accept attributes as content to perform recommendation. On the other hand, researchers also try to extend an existing collaborative filtering algorithm such that it leverages attribute information.

**Figure 4:**
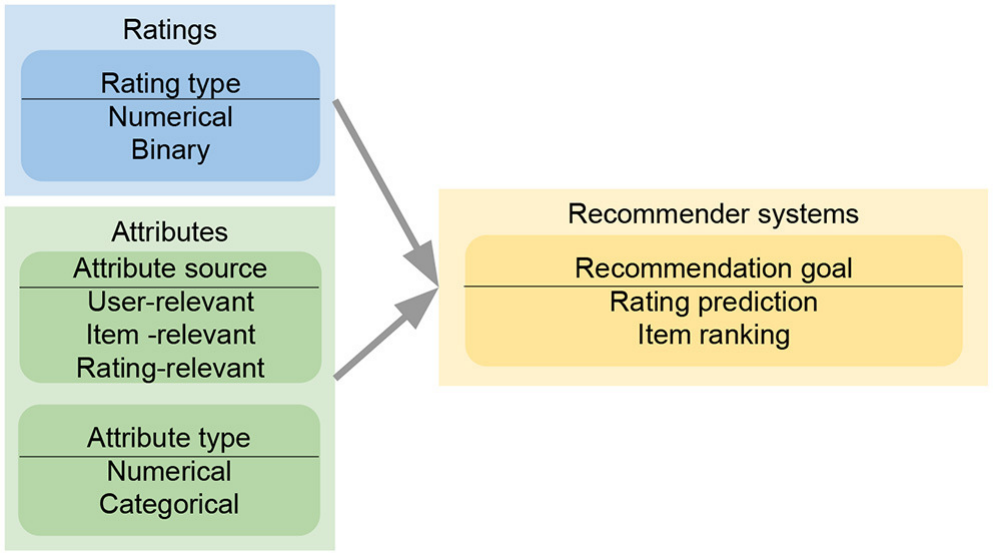
Model design flow of an attribute-aware collaborative filtering-based recommender system. When reading ratings and attributes for a proposed approach, we have to consider the sources and the types of attributes or ratings, which could affect the recommendation goals and model designs. The evaluation of a proposed recommender system depends heavily on the chosen recommendation goals.

Rather, we will focus on four important factors in the design of an attribute-aware recommender system in current research, as shown in Figure 4. They are specifically discussed from sections 3.2 to 3.5. With respect to input data, *attribute sources* determine whether an attribute vector is relevant to users, items, or ratings. For example, *age* describes a user instead of an item; *rating time* must be appended to ratings, representing when the rating event occurred. Different models impose distinct strategies to integrate attributes of specific sources. Additionally, a model may constrain *attribute types* that can be used. For instance, graph-based collaborative filtering realizations define attributes as node types, which is not appropriate for numerical attributes. *Rating type* is the factor that is emphasized by most model designers. Besides usual numerical ratings, many recommendation models concentrate on binary rating data, where the ratings represent whether users interact with items. Finally, different recommender systems emphasize different *recommendation goals*. One is to predict the ratings from users to items through minimizing the error between the predicted and real ratings. Another is to produce the ranking among items given a user instead of caring about the real rating value of a single item. We then summarize the design categories of all the surveyed papers in a table in section 3.6.

Throughout this paper, we will use $\text{X}=\left[\begin{array}{c} {\text{x}}_{1}{\text{x}}_{2}\ldots {\text{x}}_{N} \end{array}\right]\in {\mathbb{R}}^{K\times N}$ to denote the attribute matrix, where each column ***x***_*i*_ represents a *K*-dimensional attribute vector of entity *i*. Here, an entity can refer to a user, an item, or a rating, determined by attribute sources (discussed in section 3.2). If attributes are limited categorically, then ***X*** ∈ {0, 1}^*K*×*N*^ can be represented by one-hot encoding (discussed in section 3.3). Note that our survey does not include models designed specifically for a certain type of attribute but rather covers models that are general enough to accept different types of attributes. For example, Collaborative Topic Regression (CTR) (Wang and Blei, 2011) extends matrix factorization with Latent Dirichlet Allocation (LDA) to import text attributes. Social Regularization (Ma et al., 2011a) specifically utilizes user social networks to regularize the learning of matrix factorization. Neither model is included since they are not general enough to deal with general attributes.

### 3.2. Sources of Attributes

Attributes usually come from a variety of sources. Typically, *side information* refers to the attributes appended to users or items. In contrast, keyword *contexts* indicate the attributes relevant to ratings. Ratings from the same user can be attached to different contexts, such as “locations where users rate items.” Recommendation models considering rating-relevant attributes are usually called *context-aware recommender systems*. Although contexts in some papers could include user-relevant or item-relevant ones, in this paper, we tend to be precise and use the term *contexts* only for rating-relevant attributes.

Sections 3.2.1 and 3.2.2, respectively introduce different attribute sources. It is worth mentioning our observation as follows. Even though some of the models we surveyed demand side information while others require context information, we discover that the two sets of attributes can be represented in a unified manner and thus that both types of models can be applied. We will discuss such unified representation in sections 3.2.3 and 3.2.4.

#### 3.2.1. Side Information: User-Relevant or Item-Relevant Attributes

In the surveyed papers, *side information* could refer to user-relevant attributes, item-relevant attributes, or both. User-relevant attributes determine the characteristics of a user, such as “age,” “gender,” “education,” etc. In contrast, item-relevant attributes describe the properties of an item, like “movie running time,” “product expiration data,” etc. Below, we discuss user-relevant attributes, but all the statements can be applied to item-relevant attributes. Given user-relevant attributes, we can express them with matrix $\text{X}\in {\mathbb{R}}^{K\times N_{u}}$, where *N*_*u*_ is the number of users. Each column of ***X*** corresponds to *K* attribute values of a specific user. The most important characteristic of user-relevant attributes is that they are *assumed unchanged* with the rating process of a user. For example, every rating from the same user shares the identical user-relevant attribute “age.” In other words, even without any ratings from a user in collaborative filtering, the user's rating behaviors on items could be still extracted from other users that have similar user-relevant attribute values. Attribute-aware recommender systems that address the cold-start user problems (i.e., there are few ratings of a user) typically adopt user-relevant attributes as their auxiliary information under collaborative filtering. The attribute leverage methods are presented in section 4.

Readers may ask why user-relevant attributes and item-relevant attributes are not distinguished. Our observations during this survey indicate that most of the recommendation approaches have symmetric model designs for users and items. In matrix factorization-based methods, rating matrix ***R*** is factorized into two matrices ***W*** and ***H***, referring to user and item latent factors, respectively. However, matrix factorization does not change its learning results if we exchange the rows and columns of ***R***. Despite the exchange of rows and columns, ***W*** and ***H*** just exchange what they learn from ratings: ***W*** for items but ***H*** for users.

On the basis of the above conclusions, some of the related work could be further extended, in our opinion. If one attribute-aware recommender system claims to be designed only for user-relevant attributes, then readers could use a symmetric model design for item-relevant attributes to obtain a more general model.

#### 3.2.2. Contexts: Rating-Relevant Attributes

Collaborative filtering-based recommender systems usually define ratings as *the* interaction between users and items, though it is likely to have more than one interaction. Since ratings are still the focus of recommender systems, other types of interactions, or rating-relevant attributes, are called *contexts* in related work. For example, the “time” and the “location” that a user rates an item are recorded with the occurrence of the rating behavior. Rating-relevant attributes change with rating behaviors, and thus they could offer auxiliary data on why a user decides to give a rating to an item. Moreover, rating-relevant attributes could capture the rating preference change of a user. If we have time information appended to ratings, then attribute-aware recommender systems could discover users' preferences at different times.

The format of rating-relevant attributes is potentially more flexible than that of user-relevant or item-relevant ones. In section 4.3, we will introduce a factorization-based generalization of matrix factorization. In this class of attribute-aware recommender systems, even the user and item latent factors are not required to predict ratings; mere rating-relevant attributes can do it, using their corresponding latent factor vectors.

#### 3.2.3. Converting Side Information to Contexts

Most attribute-aware recommender systems choose to leverage one of the attribute sources. Some proposed approaches specifically incorporate user- or item-relevant attributes, while others are designed for rating-relevant attributes only. It seems that existing approaches should be applied according to which attribute sources they use. However, we argue that the usage of attribute-aware recommender systems could be independent of attribute sources if we convert them to each other using a simple method.

Let $\text{X}\in {\mathbb{R}}^{K_{X}\times N_{u}}$ be the user-relevant attribute matrix, where each column ${\text{x}}_{u}\in {\mathbb{R}}^{K_{X}}$ is the attribute set of user *u*. Similarly, let $Y\in {\mathbb{R}}^{K_{Y}\times N_{i}},Z\in {\mathbb{R}}^{K_{Z}\times N_{r}}$ be the matrices of item-relevant attributes and rating-relevant attributes, respectively. Note that a column index of matrix ***Z*** is denoted by π(*u, i*), which is associated with user *u* and item *i*. A simple concatenation with respect to users and items can achieve the goal of expressing ***X*** or ***Y*** as ***Z***, as shown below:

(11) $$\begin{array}{l} z_{\pi \left(u,i\right)}^{\prime }=\left[\begin{array}{c} z_{\pi \left(u,i\right)}\\ x_{u}\\ y_{i} \end{array}\right]\in {\mathbb{R}}^{K_{Z}+K_{X}+K_{Y}}. \end{array}$$

Equation (11) implies that we just extend the current rating-relevant attributes ***z***_π(*u,i*)_ to ${\text{z}}_{\pi \left(u,i\right)}^{\prime }$ using the attributes ***x***_*u*_, ***y***_*i*_ from corresponding users or items. If training data do not consist of ***z***_π(*u,i*)_, ***x***_*u*_ or ***y***_*i*_, we can eliminate the notations on the right-hand side of (11). Advanced attribute selection or dimensionality reduction methods could extract effective dimensions in ${\text{z}}_{\pi \left(u,i\right)}^{\prime }$, but further improvement is beyond our scope. If missing attribute values exist in ${\text{z}}_{\pi \left(u,i\right)}^{\prime }$, then we suggest directly filling these attributes with 0. Please refer to section 3.2.4 for our reasoning.

#### 3.2.4. Converting Contexts to Side Information

Following the topic in section 3.2.3, the reader may be curious about how to reversely convert rating-relevant attributes to user- or item-relevant ones. In the following paragraphs, we adopt the same notations as in section 3.2.3. Due to symmetric designs for ***X*** and ***Y***, we demonstrate only the conversion from ***Z*** to ***X***. Concatenation is still the simplest way to express ***Z*** as a part of ***X***:

(12) $$\begin{array}{l} x_{u}^{\prime }={\left[\begin{array}{c} x_{u}^{⊤}{\;z}_{\pi \left(u,1\right)}^{⊤}{\;z}_{\pi \left(u,2\right)}^{⊤}\ldots {\;z}_{\pi \left(u,i\right)}^{⊤}\ldots {\;z}_{\pi \left(u,N_{i}\right)}^{⊤} \end{array}\right]}^{⊤}\in {\mathbb{R}}^{K_{X}+K_{Z}N_{i}}. \end{array}$$

All the rating-relevant attributes ***z***_(*u*,1)_, ***z***_(*u*,2)_, …, ***z***_(*u*,*N*_*i*_)_ from *N*_*i*_ items must be associated with user *u*. ***x***_*u*_ is thus extended to ${\text{x}}_{u}^{\prime }$ by appending these attributes. Note that there are a large number of missing attributes on the right-hand side of (12), since most items are never rated by user *u* in real-world data. Eliminating missing ***z***_π(*u,i*)_, as what we do in section 3.2.3, reveals different dimensions between two user-relevant attributes ${\text{x}}_{u}^{\prime },{\text{x}}_{v}^{\prime }$. To our knowledge, there is no user-relevant attribute-aware recommender system allowing individual dimensions of user-relevant attributes.

Readers can use attribute imputation approaches to remove missing values in ${\text{x}}_{u}^{\prime }$. However, we argue that simply filling missing elements with 0 is sufficient for attribute-aware recommender systems. We explain our reasons through the observations in section 3.3. For numerical attributes, (13)–(15) show the various attribute modeling methods. If attributes ***X*** are mapped through function *f* like (13) or (15), then zero attributes in *f* will cause no mapping effect (except constant intercept of *f*). If attributes ***X*** are fitted by latent factors onto function *f* such as (14), then typically in the objective design, we can skip the objective computation of missing attributes. As for categorical attributes, we exploit one-hot encoding to represent them with numerical values. Categorical attributes can then be handled as numerical attributes.

### 3.3. Attribute Types

In most cases, attribute-aware recommender systems accept a real-value attribute matrix ***X***. However, we notice that some attribute-aware recommender systems require attributes to be categorical, which is typically represented by binary encoding. Specifically, this approach demands a binary attribute matrix where attributes of value 1 are modeled as discrete latent information in some way. A summary of the two types of attributes is given in sections 3.3.1 and 3.3.2.

It is trivial to put one-hot categorical attributes into numerical attribute-aware recommender systems, since binary values {0, 1} ⊂ ℝ. Nonetheless, putting numerical attributes into categorical attribute-aware recommendation systems runs the risk of losing attribute information (e.g., quantization processing).

#### 3.3.1. Numerical Attributes

In our paper, numerical attributes refer to the set of real-valued attributes, i.e., attribute matrix ***X*** ∈ ℝ^*K*×*N*^. We also classify integer attributes (like movie ratings {1, 2, 3, 4, 5}) to numerical attributes. Most of the relevant papers model numerical attributes as their default inputs in recommender systems, as is common in machine learning approaches.

There are three common model designs through which numerical attributes ***X*** affect recommender systems. First, we can map ***X*** to latent factor space by function *f*_***θ***_ with parameters ***θ***, and then fit the corresponding user or item latent factor vectors:

(13) $$\begin{array}{l} \underset{\theta ,W}{\mathrm{argmin}}||f_{\theta }\left(X\right)-W||\text{or }W=f_{\theta }\left(X\right)\text{ for user-relevant attributes},\\ \underset{\theta ,H}{\mathrm{argmin}}||f_{\theta }\left(X\right)-H||\text{or }H=f_{\theta }\left(X\right)\text{ for item-relevant attributes}. \end{array}$$

Second, like the reverse of (13), we define a mapping function *f*_***θ***_ such that mapped values from user or item latent factors can be close to observed attributes:

(14) $$\begin{array}{l} \underset{\theta ,W}{\mathrm{argmin}}||f_{\theta }\left(W\right)-X||\text{or }X=f_{\theta }\left(W\right)\text{ for user-relevant attributes},\\ \underset{\theta ,H}{\mathrm{argmin}}||f_{\theta }\left(H\right)-X||\text{or }X=f_{\theta }\left(H\right)\text{ for item-relevant attributes}. \end{array}$$

Finally, numerical attributes can be put into function *f*_***θ***_, which is independent of existing user or item latent factors in matrix factorization:

(15) $$\begin{array}{l} \underset{\theta ,W,H}{\mathrm{argmin}}||f_{\theta }\left(X\right)+W^{⊤}H-R||. \end{array}$$

Equations (13) and (14) are typically seen in user-relevant or item-relevant attributes, while rating-relevant attributes are often put into (15)-like formats. However, we emphasize that attribute-aware recommender systems are not limited to these three model designs.

#### 3.3.2. Categorical Attributes

The values of a numerical attribute are ordered, while, on the other hand, the values of a categorical attribute show no ordered relations with each other. Given a categorical attribute Food ∈ {Rice, Noodles, Other}, the meanings of the values do not imply which one is larger than the other. Thus, it is improper to give categorical attributes ordered dummy variables, like Rice = 0, Noodles = 1, Other = 2, which could incorrectly imply Rice < Noodles < Other, misleading machine learning models. The most common solution to categorical attribute transformation is *one-hot encoding*. We generate *d*-dimensional binary attributes that correspond to the *d* values of a categorical attribute. Each of the *d* binary attributes indicates the current value of a categorical attribute. For example, we express attribute Food ∈ {{1, 0, 0}, {0, 1, 0}, {0, 0, 1}}. These correspond to the original values {Rice, Noodles, Other}. Since the values of the categorical attribute are unique, the mapped binary attributes contain only a 1, and others, 0. Once all the categorical attributes are converted to one-hot encoding expressions, we can apply them to existing numerical attribute-aware recommender systems.

Certain relevant papers are suitable for, or even adversely limited to, categorical attributes. Heterogeneous graph-based methods (section 4.4) add new nodes (e.g., Rice, Noodles, Other) to represent the values of categorical attributes. Following the concept of latent factor in matrix factorization, some methods propose to assign each categorical attribute value a low-dimensional latent factor vector (e.g., each of Rice, Noodles, Other has a latent factor vector ***w*** ∈ ℝ^*K*^). These vectors are then jointly learned with classical user or item latent factors in attribute-aware recommender systems.

### 3.4. Rating Types

Although we always define the term *ratings* as the interactions between users and items in this paper, some previous works claim a difference between explicit opinions and implicit feedback. Taking the dataset MovieLens, for example, a user gives a rating value in {1, 2, 3, 4, 5} toward an item. The value denotes the *explicit opinion*, which quantifies the preference of the user for that item. How recommendation methods handle such type of ratings will be introduced in section 3.4.1.

Even though modeling explicit opinions is more beneficial for future recommendation, such data is more difficult to gather from users. Users may hesitate to show their preferences due to privacy considerations, or they may not be willing to spend time labeling explicit ratings. Instead, recommender systems are more likely to collect *implicit feedback*, such as user browsing logs. Such datasets record a series of binary values, each of which implies whether a user ever saw an item. User preferences behind implicit feedback assume that the items seen by a user must be more preferred by the user than those items that have never been seen. We discuss this type of rating in detail in section 3.4.2.

Some numerical rating data are controversial, like “the number of times a user clicks on the hyperlink to visit the page of an item.” Some of the related work may define such data as implicit feedback because the number of clicks is not equivalent to explicit user preferences. However, in this paper, we still identify them as explicit opinions. With respect to model designs, related recommendation approaches do not differentiate such data.

#### 3.4.1. Explicit Opinions: Numerical Ratings

A numerical rating matrix *r* ∈ ℝ expresses users' opinions on items. Actually, numerical ratings in real-world scenarios are often represented by positive integers, such as MovieLens ratings *r* ∈ {1, 2, 3, 4, 5}. Though there are no explicit statements in related work, we suppose that a higher rating implies a more positive opinion.

Since, in most datasets, the gathered rating values are positive, there could be an unbiased learning problem. Matrix factorization could not learn the rating bias due to the non-zero mean of ratings E(*r*) ≠ 0. Specifically, in vanilla matrix factorization, we have regularization terms $||\text{W}{||}_{F}^{2}$ and $||\text{H}{||}_{F}^{2}$ for user and item latent factor matrix ***W***, ***H***. That is, we require the expected value E(***W***) = E(***H***) = **0** from the viewpoint of corresponding normal distributions. Given rating *r*_*ui*_ of user *u* to item *i*, and assuming the independence of ***W***, ***H*** as probabilistic matrix factorization does, we obtain the expected value of rating estimate $\text{E}\left({\hat{r}}_{ui}\right)=\text{E}\left({\text{w}}_{u}^{⊤}{\text{h}}_{i}\right)=0\;\forall \;\left(u,i\right)$, which cannot closely fit true ratings if E(*r*_*ui*_) ≠ 0. Biased matrix factorization can alleviate the problem by absorbing the non-zero mean with additional bias terms. Besides, we are able to normalize all the ratings (subtract the rating mean from every rating) to make matrix factorization prediction unbiased. Real-world numerical ratings also have finite maximum and minimum values. Some recommendation models choose to normalize the ratings to range *r* ∈ [0, 1] and then constrain the range of rating estimate $\text{sig}\left(\hat{r}\right)\in \left(0,1\right)$ using the sigmoid function $\text{sig}\left(x\right)=\frac{1}{1+\exp\left(-x\right)}$.

#### 3.4.2. Implicit Feedback: Binary Ratings

Today, more and more researchers are interested in the scenario of binary ratings *r* ∈ {0, 1} (i.e., implicit feedback), since such rating data are more accessible, for instance, “whether a user browsed the information about an item.” Online services do not require users to give explicit numerical ratings, which are often harder to gather than binary ones.

We observe only positive ratings *r* = 1; negative ratings *r* = 0 do not exist in training data. Taking browsing logs as an example, the data include the items that are browsed by a user (i.e., positive examples). Items not being in the browsing data could imply that they are either absolutely unattractive (*r* = 0) or just unknown (*r* ∈ {0, 1}) to the user. *One-class collaborative filtering* methods are proposed to address the problem. Such methods often claim two assumptions:

- An item must be attractive to a user (*r* = 1) as long as the user has seen the item.
- Since we cannot distinguish between the two reasons (absolutely unattractive or just unknown) why an item is unseen, such methods suppose that all the unseen items are less attractive (*r* = 0). However, the number of unseen items is practically much larger than that of seen items. To alleviate the problem of learning bias toward *r* = 0 together with learning speed, we exploit *negative sampling*, which sub-samples partial unseen ratings for training.

To build an objective function satisfying the above assumptions, we can choose either pointwise learning (section 3.5.1) or pairwise learning (section 3.5.2). The Area Under the ROC Curve (AUC), Normalized Discounted Cumulative Gain (NDCG), Mean Average Precision (MAP), precision, and recall are often used to justify the quality of recommender systems for binary ratings.

### 3.5. Recommendation Goals

Any recommender system needs human intervention to set up a training goal. Since collaborative filtering-based recommender systems rely on ratings, the most straightforward goal is to infer what rating will be given by a user for an unseen item, called *rating prediction*. If the ratings of every item can be accurately predicted, then for any user, a recommender system can just sort and recommend items based on highest predicted rating. In machine learning, such a goal for model-based recommender systems can be described as *pointwise learning*. That is, given a user-item pair, a pointwise learning recommendation model directly minimizes the error of predicted ratings and true ones. The related mathematical details are presented in section 3.5.1.

However, in general, our ultimate goal is to recommend unseen items to users without being concerned about how these items are rated. All unseen items in pointwise learning are finally ranked in descending order of their ratings. In other words, what we truly care about is the order of ratings, but not the true rating values. Also, some research papers figure out that a low error of rating prediction is not always equivalent to a high quality of recommended item lists. Recent model-based collaborative filtering models have begun to set optimization goals of *item ranking*. That is, for the same user, such models maximize the differences between high-rated items and low-rated ones in training data. The implementation of item ranking includes *pairwise learning and listwise learning* in machine-learning domains. Both learning ideas try to compare the potentially related ranks between at least two items for the same user. section 3.5.2 will present how to define optimization criteria for item ranking.

#### 3.5.1. Rating Prediction: Pointwise Learning

In the training stage, given a ground-truth rating *r*, a recommender system needs to make a rating estimate $\hat{r}$ that is expected to predict *r*. Model-based collaborative filtering methods (e.g., matrix factorization) build an objective function to be optimized (either maximization or minimization) for recommendation goals. For numerical ratings *r* ∈ ℝ (section 3.4.1) of users *u* to items *i*, we can minimize the error between the ground truth and the estimate as follows:

(16) $$\begin{array}{l} \underset{\hat{r}}{\mathrm{argmin}}\underset{\left(u,i\right)|r_{ui}\in \delta \left(R\right)}{\sum }{\left({\hat{r}}_{ui}-r_{ui}\right)}^{2},\\ \underset{\hat{r}}{\mathrm{argmin}}\underset{\left(u,i\right)|r_{ui}\in \delta \left(R\right)}{\sum }{\left(\text{sig}\left({\hat{r}}_{ui}\right)-r_{ui}\right)}^{2}. \end{array}$$

δ(***R***) is the set of training ratings, which are the non-missing entries in rating matrix ***R***. As mentioned in section 3.4.1, if ground-truth ratings *r* are normalized to [0, 1] in data pre-processing, then in (16) we can put sigmoid function $\text{sig}\left(x\right)=\frac{1}{1+\exp\left(-x\right)}\in \left(0,1\right)$ onto rating estimate $\hat{r}$ that fits *r* more closely. With respect to probability, (16) is equivalent to maximizing normal likelihood:

(17) $$\begin{array}{l} \underset{\hat{r}}{\text{argmax}}\underset{\left(u,i\right)|r_{ui}\in \delta \left(R\right)}{\prod }N\left(r_{ui}|\mu ={\hat{r}}_{ui},\sigma^{2}\right)\\ \underset{\hat{r}}{\text{argmax}}\underset{\left(u,i\right)|r_{ui}\in \delta \left(R\right)}{\prod }N\left(r_{ui}|\mu =\text{sig}\left({\hat{r}}_{ui}\right),\sigma^{2}\right) \end{array}$$

where N is the probability density function of a normal distribution with mean $\mu =\hat{r}$ and variance σ^2^ is a predefined uncertainty between *r* and $\hat{r}$. Taking (−log) on (17) will give (16). Evidently the rating prediction problem can be addressed by regression models over ratings ***R*** with both (16) and (17).

For binary ratings *r* ∈ {0, 1} (section 3.4.2), other than (16) with the sigmoid function, such data can also be modeled as a binary classification problem. Specifically, we model *r* = 1 as the positive set and *r* = 0 as the negative set. A logistic regression (or Bernoulli likelihood) is then built for rating prediction:

(18) $$\begin{array}{l} \underset{\hat{r}}{\text{argmax}}\underset{\left(u,i\right)|1=r_{ui}\in \delta \left(R\right)}{\prod }\mathrm{Pr}\left({\hat{r}}_{ui}=1\right)\underset{\left(u,i\right)|0=r_{ui}\in \delta \left(R\right)}{\prod }\mathrm{Pr}\left({\hat{r}}_{ui}=0\right)\\ \;=\underset{\hat{r}}{\text{argmax}}\underset{\text{Positive set}}{\underset{︸}{\underset{\left(u,i\right)|1=r_{ui}\in \delta \left(R\right)}{\prod }\text{sig}\left({\hat{r}}_{ui}\right)}}\underset{\text{Negative set}}{\underset{︸}{\underset{\left(u,i\right)|0=r_{ui}\in \delta \left(R\right)}{\prod }\left(1-\text{sig}\left({\hat{r}}_{ui}\right)\right)}}. \end{array}$$

The optimizations of (16) and (17) are based on an evaluation metric: Root Mean Squared Error (RMSE), whose formal definition is as follows:

(19) $$\begin{array}{l} \text{RMSE}=\sqrt{\frac{1}{\left|\delta \left(R\right)\right|}\underset{\left(u,i\right)|r_{ui}\in \delta \left(R\right)}{\sum }{\left({\hat{r}}_{ui}-r_{ui}\right)}^{2}}. \end{array}$$

For convenience of optimization, the regression models eliminate the root function from RMSE, essentially optimizing the MSE. Since the root function is monotonically increasing, minimizing MSE is equivalent to minimizing RMSE (19).

Even though a recommender system elects to optimize (18), the binary classification also attempts to minimize RMSE, except that rating estimate $\hat{r}$ is replaced with sigmoid-applied version $\text{sig}\left(\hat{r}\right)$. Observing the maximization of (18), we obtain a conclusion: $\text{sig}\left(\hat{r}\right)\to 1$ as *r* = 1, or $\text{sig}\left(\hat{r}\right)\to 0$ as *r* = 0. In other words, (18) tries to minimize the error between $\text{sig}\left(\hat{r}\right)\in \left(0,1\right)$ and *r* ∈ {0, 1}, which has the same optimization goal as RMSE (19).

#### 3.5.2. Item Ranking: Pairwise Learning and Listwise Learning

This class of recommendation goal requires a model to correctly rank two items in the training data, even though the model could inaccurately predict the value of a single rating. Since recommender systems are more concerned about item ranking for the same user *u* than ranking for different users, existing approaches sample item pairs (*i, j*) where *r*_*ui*_ > *r*_*uj*_, given fixed user *u* (i.e., item *i* is ranked higher than item *j* for user *u*), and then let rating estimate pair $\left({\hat{r}}_{ui},{\hat{r}}_{uj}\right)$ learn to rank the two items with ${\hat{r}}_{ui}>{\hat{r}}_{uj}$. In particular, we can use the sigmoid function $\text{sig}\left(x\right)=\frac{1}{1+\exp\left(-x\right)}$ to model the probabilities in the pairwise comparison likelihood:

(20) $$\begin{array}{l} \underset{\hat{r}}{\text{argmax}}\prod_{(u,i,j){|}_{\;r_{ui}>r_{uj}}^{\{r_{ui},r_{uj}\}\subseteq \delta (R),}}\mathrm{Pr}\left({\hat{r}}_{ui}>{\hat{r}}_{uj}\right)\\ \;=\underset{\hat{r}}{\text{argmax}}\prod_{(u,i,j){|}_{\;r_{ui}>r_{uj}}^{\{r_{ui},r_{uj}\}\subseteq \delta (R),}}\text{sig}({\hat{r}}_{ui}>{\hat{r}}_{uj}). \end{array}$$

Taking (−log) on objective function (20) will give the log-loss function. Bayesian Personalized Ranking (BPR) (Rendle et al., 2009) first investigated the usage and the optimization of (20) for recommender systems. BPR shows that (20) maximizes a differentiable smoothness of the evaluation metric Area Under the ROC Curve (AUC):

(21) $$\text{AUC}=\frac{1}{T}\sum_{(u,i,j){|}_{\;r_{ui}>r_{uj}}^{\{r_{ui},r_{uj}\}\subseteq \delta (R),}}\text{I}\left({\hat{r}}_{ui}>{\hat{r}}_{uj}\right),$$

where *T* is the number of training instances {(*u, i, j*) ∣ {*r*_*ui*_, *r*_*uj*_} ⊆ δ(***R***), *r*_*ui*_ > *r*_*uj*_}. I(*x*) ∈ {0, 1} denotes an indicator function whose output is 1 if and only if condition *x* is judged true. We show the connection between (20) and (21) below:

(22) $$\begin{array}{l} \underset{\hat{r}}{\text{argmax}}\;(21)=\underset{\hat{r}}{\text{argmax}}\sum_{(u,i,j){|}_{\;r_{ui}>r_{uj}}^{\{r_{ui},r_{uj}\}\subseteq \delta (R),}}\text{I}\left({\hat{r}}_{ui}-{\hat{r}}_{uj}>0\right)\\ \;\approx \underset{\hat{r}}{\text{argmax}}\sum_{(u,i,j){|}_{\;r_{ui}-r_{uj}>0}^{\{r_{ui},r_{uj}\}\subseteq \delta (R),}}\text{sig}\left({\hat{r}}_{ui}-{\hat{r}}_{uj}\right)\\ \;\approx \underset{\hat{r}}{\text{argmax}}\sum_{(u,i,j){|}_{\;r_{ui}-r_{uj}>0}^{\{r_{ui},r_{uj}\}\subseteq \delta (R),}}\text{log sig}\left({\hat{r}}_{ui}-{\hat{r}}_{uj}\right)\\ \;=\underset{\hat{r}}{\text{argmax }\log}\prod_{(u,i,j){|}_{\;r_{ui}-r_{uj}>0}^{\{r_{ui},r_{uj}\}\subseteq \delta (R),}}\text{sig}\left({\hat{r}}_{ui}-{\hat{r}}_{uj}\right). \end{array}$$

Under the condition of argmax, we approximate non-differentiable indicator function I(*x*) by differentiable sigmoid function sig(*x*). The maximization of (22) is equivalent to optimizing (20) due to the monotonically increasing logarithmic function. AUC evaluates whether all the predicted item pairs follow the ground-truth rating comparisons in the whole item list. Our observations indicate that most of the reviewed approaches based on item ranking build their objective functions with AUC optimization. There are other choices of optimization functions to approximately maximize AUC, such as hinge loss:

(23) $$\underset{\hat{r}}{\text{argmin}}\sum_{(u,i,j){|}_{\;r_{ui}>r_{uj}}^{\{r_{ui},r_{uj}\}\subseteq \delta (R),}}\text{max}\left\{0,{\hat{r}}_{uj}-{\hat{r}}_{ui}\right\}.$$

In the domain of top-*N* recommendation, the item order outside top-*N* ranks is unimportant for recommender systems. Maximizing AUC could fail to recommend items, since AUC gives the same penalty to all items. That is, a recommender system could gain high AUC when it accurately ranks the bottom-*N* items, but it is not beneficial for real-world recommendation, since a user only pays attention to the top-*N* items. Listwise evaluation metrics like Mean Reciprocal Rank (MRR), Normalized Discounted Cumulative Gain (NDCG), and Mean Average Precision (MAP) are proposed to give different penalty values to item ranking positions. There have been attempts to optimize differential versions of the above metrics, such as CliMF (Shi et al., 2012b), SoftRank (Taylor et al., 2008), and TFMAP (Shi et al., 2012a).

Our observations of the surveyed papers indicate that recommender systems reading binary ratings (section 3.4.2) prefer to optimize an item-ranking objective function. Compared with numerical ratings (section 3.4.1), a single binary rating reveals less information on a user's absolute preference. Pairwise learning methods can capture more information by modeling a user's relative preferences, because the number of rating pairs *r*_*ui*_ = 1 > 0 = *r*_*uj*_ is more than the number of ratings for each user.

### 3.6. Summary of Related Work

Having introduced the above categories for attribute-aware recommender systems, we list which category each publication we have surveyed belongs to in Tables 2–4. We trace back 10 years to summarize the recent trends in attribute-aware recommender systems.

**Table 2:** List of model categories.

**Model**	**Year**	**Attri. Source (3.2)**			**Attri. Type (3.3)**		**Rating type (3.4)**		**Recom. Goal (3.5)**	
		**User**	**Item**	**Rating**	**Num**.	**Cat**.	**Num**.	**Bin**.	**Pred**.	**Rank**.
		**(3.2.1)**	**(3.2.1)**	**(3.2.2)**	**(3.3.1)**	**(3.3.2)**	**(3.4.1)**	**(3.4.2)**	**(3.5.1)**	**(3.5.2)**
CMF (Singh and Gordon, 2008)	2008	✓	✓		✓		✓		✓	
TBM (Gunawardana and Meek, 2008)	2008		✓		✓			✓	✓	
WNMCTF (Yoo and Choi, 2009)	2009	✓	✓			✓	✓		✓	
CAR-AUC (Shin et al., 2009)	2009			✓		✓		✓	✓	
Multi. Recom.^a^ (Weng et al., 2009)	2009			✓		✓	✓		✓	
RLFM (Agarwal and Chen, 2009)	2009	✓	✓	✓	✓		✓		✓	
Unified Boltz (Gunawardana and Meek, 2009)	2009		✓		✓			✓	✓	
Matchbox (Stern et al., 2009)	2009	✓	✓	✓	✓		✓	✓	✓	
BMFSI (Porteous et al., 2010)	2010	✓	✓		✓		✓		✓	
wAMAN.^b^ (Li et al., 2010a)	2010	✓			✓			✓	✓	
CACF (Lee et al., 2010)	2010			✓	✓			✓	✓	
PLRM (Li et al., 2010b)	2010	✓	✓			✓	✓		✓	
LAFM (Gantner et al., 2010)	2010	✓	✓		✓			✓		✓
GPMF (Shan and Banerjee, 2010)	2010		✓			✓	✓		✓	
LFL (Menon and Elkan, 2010)	2010			✓	✓		✓		✓	
TF (Karatzoglou et al., 2010)	2010			✓		✓	✓		✓	
GWNMTF (Gu et al., 2010)	2010	✓	✓		✓		✓		✓	
DPMF (Adams et al., 2010)	2010	✓	✓		✓		✓		✓	
SoRec (Ma et al., 2011b)	2011	✓	✓		✓		✓		✓	
UGPMF (Du et al., 2011)	2011	✓			✓			✓		✓
BMCF (Yoo and Choi, 2011)	2011	✓	✓		✓		✓		✓	
MCRI (Fang and Si, 2011)	2011	✓	✓		✓			✓	✓	
Hybrid.^c^ (Menon et al., 2011)	2011			✓	✓			✓	✓	
YMR (Koenigstein et al., 2011)	2011	✓	✓			✓	✓		✓	
CAMF (Baltrunas et al., 2011)	2011			✓		✓	✓		✓	
GFREC (Lee et al., 2011)	2011			✓		✓		✓		✓
FM (Rendle et al., 2011)	2011			✓	✓		✓		✓	
FIP (Yang et al., 2011)	2011	✓	✓		✓			✓	✓	
iTALS (Hidasi and Tikk, 2012)	2012			✓		✓		✓	✓	
HVBMCF (Yoo and Choi, 2012)	2012	✓	✓		✓		✓		✓	
LCR (Weston et al., 2012)	2012		✓		✓			✓		✓
HierIntegModel (Lu et al., 2012)	2012		✓			✓	✓		✓	
SVDFeature (Chen et al., 2012)	2012	✓	✓	✓	✓		✓		✓	
SSLIM (Ning and Karypis, 2012)	2012		✓		✓			✓	✓	
KPMF (Zhou et al., 2012)	2012	✓	✓		✓		✓		✓	
TFMAP (Shi et al., 2012a)	2012			✓		✓		✓		✓
CCMF (Bouchard et al., 2013)	2013	✓	✓		✓		✓		✓	
GFMF (Chen et al., 2013)	2013	✓	✓		✓		✓		✓	
KBMF (Gönen et al., 2013)	2013	✓	✓		✓		✓		✓	
HBMFSI (Park et al., 2013)	2013	✓	✓		✓		✓		✓	
DACR (Safoury and Salah, 2013)	2013	✓				✓	✓		✓	
Maxide (Xu et al., 2013)	2013	✓	✓		✓		✓		✓	
MF-EFS (Koenigstein and Paquet, 2013)	2013		✓		✓			✓	✓	
HeteroMF (Jamali and Lakshmanan, 2013)	2013	✓	✓		✓		✓		✓	
SoCo (Liu and Aberer, 2013)	2013			✓	✓	✓	✓		✓	

*Numbers in parentheses refer to the sections of the current paper giving category elaborations. All the model names come from the proposing publications, except that we use title abbreviations if the authors do not name their approaches. Long model names are given in the footnotes*.

a *Multidimensional Recommendation*.

b *wAMANWithSchKW*.

c *Hybrid+LogReg++*.

**Table 3:** List of model categories.

**Model**	**Year**	**Attri. Source (3.2)**			**Attri. Type (3.3)**		**Rating type (3.4)**		**Recom. Goal (3.5)**	
		**User**	**Item**	**Rating**	**Num**.	**Cat**.	**Num**.	**Bin**.	**Pred**.	**Rank**.
		**(3.2.1)**	**(3.2.1)**	**(3.2.2)**	**(3.3.1)**	**(3.3.2)**	**(3.4.1)**	**(3.4.2)**	**(3.5.1)**	**(3.5.2)**
C-CTR-SMF2 (Chen et al., 2014)	2014	✓	✓	✓	✓		✓		✓	
VBMFSI-CA (Kim and Choi, 2014)	2014	✓	✓		✓		✓		✓	
IMC (Natarajan and Dhillon, 2014)	2014	✓	✓		✓			✓	✓	
CARS^2^ (Shi et al., 2014a)	2014	✓	✓		✓		✓		✓	✓
LLR (Ji et al., 2014)	2014		✓			✓	✓		✓	
GBFM (Cheng et al., 2014)	2014			✓	✓		✓		✓	
SCF (Sedhain et al., 2014)	2014	✓				✓	✓		✓	
LCE (Saveski and Mantrach, 2014)	2014		✓		✓		✓		✓	
CSEL (Zhang et al., 2014)	2014	✓	✓			✓	✓		✓	
GPFM (Nguyen et al., 2014)	2014		✓	✓		✓	✓		✓	✓
NCRPD-MF (Hu et al., 2014)	2014		✓	✓	✓		✓		✓	
HeteRec (Yu et al., 2014)	2014		✓			✓		✓		✓
CAPRF (Gao et al., 2015)	2015	✓	✓	✓			✓			✓
mSDA-CF (Li et al., 2015)	2015	✓	✓		✓		✓		✓	
BIMC (Shin et al., 2015)	2015	✓	✓		✓			✓	✓	
Convex FM (Blondel et al., 2015)	2015			✓	✓		✓		✓	
CDL (Wang et al., 2015)	2015		✓		✓			✓	✓	
LightFM (Kula, 2015)	2015	✓	✓			✓		✓	✓	
DCT (Barjasteh et al., 2015)	2015	✓	✓		✓		✓		✓	
GFF (Hidasi, 2015)	2015			✓		✓		✓	✓	
CALR (Liu and Wu, 2015)	2015	✓	✓		✓		✓		✓	
VBPR (He and McAuley, 2016)	2016		✓		✓			✓		✓
GFF (Hidasi and Tikk, 2016)	2016			✓		✓		✓	✓	
PNFM (Blondel et al., 2016)	2016			✓	✓		✓		✓	
TCRM (Kasai and Mishra, 2016)	2016			✓		✓	✓		✓	
PCFSI (Zhao et al., 2016)	2016		✓		✓		✓		✓	
CKE (Zhang et al., 2016)	2016		✓		✓			✓		✓
CRAE (Wang et al., 2016)	2016		✓		✓		✓		✓	
SIMMCSI (Lu et al., 2016)	2016	✓	✓		✓		✓		✓	
DSR (Zheng et al., 2016)	2016	✓	✓			✓	✓		✓	
ALMM (Chou et al., 2016)	2016		✓		✓		✓		✓	
FFM (Juan et al., 2016)	2016	✓	✓	✓	✓			✓	✓	
ReMF (Yang et al., 2016)	2016	✓				✓	✓		✓	
TAPER (Ge et al., 2016)	2016			✓		✓		✓	✓	
LPRRM-CF (Chen et al., 2016)	2016			✓		✓	✓		✓	
HeteRS (Pham et al., 2016)	2016	✓	✓	✓		✓		✓		✓
MVM (Cao et al., 2016)	2016			✓	✓		✓		✓	
SQ (Yu et al., 2017)	2017	✓	✓		✓			✓	✓	
LoCo (Sedhain et al., 2017)	2017	✓			✓			✓	✓	
aSDAE (Dong et al., 2017)	2017	✓	✓		✓		✓		✓	
CoEmbed (Guo, 2017)	2017	✓	✓		✓			✓	✓	
HMF (Brouwer and Liò, 2017)	2017	✓	✓		✓		✓		✓	
DeepFM (Guo et al., 2017)	2017	✓	✓		✓			✓	✓	
LDRSSI (Feipeng Zhao, 2017)	2017		✓		✓			✓	✓	
CGSI (Zhou T. et al., 2017)	2017	✓	✓	✓	✓	✓	✓		✓	

*The numbers in parentheses refer to the sections of the current paper giving category elaborations. All the model names come from the proposing publications, except that we use title abbreviations if the authors do not name their approaches. Long model names are given in the footnotes*.

**Table 4:** List of model categories.

**Model**	**Year**	**Attri. Source (3.2)**			**Attri. Type (3.3)**		**Rating Type (3.4)**		**Recom. Goal (3.5)**	
		**User**	**Item**	**Rating**	**Num**.	**Cat**.	**Num**.	**Bin**.	**Pred**.	**Rank**.
		**(3.2.1)**	**(3.2.1)**	**(3.2.2)**	**(3.3.1)**	**(3.3.2)**	**(3.4.1)**	**(3.4.2)**	**(3.5.1)**	**(3.5.2)**
Func. Embed.^a^ (Chen et al., 2017)	2017	✓	✓		✓			✓	✓	✓
CVAE (Li and She, 2017)	2017		✓		✓		✓		✓	
entity2rec (Palumbo et al., 2017)	2017		✓			✓		✓		✓
NFM (He and Chua, 2017)	2017			✓	✓		✓		✓	
MFM (Lu et al., 2017)	2017			✓	✓		✓		✓	
Focused FM (Beutel et al., 2017)	2017		✓			✓	✓		✓	
GB-CENT (Zhao et al., 2017)	2017			✓	✓		✓		✓	
CML (Hsieh et al., 2017)	2017		✓		✓			✓		✓
ATRank (Zhou C.et al., 2017)	2018			✓		✓	✓		✓	
Div-HeteRec (Nandanwar et al., 2018)	2018	✓	✓	✓		✓		✓	✓	
HeteLearn (Jiang et al., 2018)	2018	✓	✓	✓		✓		✓		✓
RNNLatentCross (Beutel et al., 2018)	2018			✓	✓		✓		✓	
DDL (Zhang et al., 2018)	2018		✓			✓	✓		✓	

*The numbers in parentheses refer to the sections of the current paper giving category elaborations. All the model names come from the proposing publications, except that we use title abbreviations if the authors do not name their approaches. Long model names are given in the footnotes*.

a *Functional Embedding*.

## 4. Common Model Designs of Attribute-Aware Recommender Systems

In this section, we formally introduce the common attribute integration methods of existing attribute-aware recommender systems. If collaborative filtering approaches are modeled by user or item latent factor structures like matrix factorization, then attribute matrices become either the prior knowledge of the latent factors (section 4.1) or the generation outputs from the latent factors (section 4.2). On the other hand, some approaches used are actually generalizations of matrix factorization (section 4.3). Besides, the interactions between users and items can be recorded by a heterogeneous network, which can incorporate attributes by simply adding attribute-representing nodes (section 4.4). The major distinction of these four categories lies in the representation of the interactions of users, items, and attributes. The discriminative matrix factorization models extend the traditional MF by learning the latent representation of users or items from the input attribute prior knowledge. Generative matrix factorization further considers the distributions of attributes and learns such together with the rating distributions. Generalized factorization models view the user/item identity simply as a kind of attribute, and various models have been designed for learning the low-dimensional representation vectors for rating prediction. The last category of models propose to represent the users, items, and attributes using a heterogeneous graph, where a recommendation task can be cast into a link prediction task on the heterogeneous graph. We classify each model into these categories in Table 5.

**Table 5:** Classification of attribute-aware recommender systems.

DMF	Similarity	Adams et al., 2010; Gu et al., 2010; Li et al., 2010a; Du et al., 2011; Zhou et al., 2012; Gönen et al., 2013; Chen et al., 2014; Barjasteh et al., 2015; Yu et al., 2017
	Linear	Menon and Elkan, 2010; Porteous et al., 2010; Menon et al., 2011; He and McAuley, 2016; Zhao et al., 2016; Feipeng Zhao, 2017; Guo, 2017
	Bilinear	Agarwal and Chen, 2009; Stern et al., 2009; Li et al., 2010b; Yang et al., 2011; Chen et al., 2012; Park et al., 2013; Xu et al., 2013; Kim and Choi, 2014; Natarajan and Dhillon, 2014; Shin et al., 2015; Chou et al., 2016; Lu et al., 2016
GMF	Multiple Matrix Factorization	Singh and Gordon, 2008; Shan and Banerjee, 2010; Fang and Si, 2011; Ma et al., 2011b; Yoo and Choi, 2011; Bouchard et al., 2013; Saveski and Mantrach, 2014; Gao et al., 2015; Ge et al., 2016; Brouwer and Liò, 2017; Sedhain et al., 2017
	Deep Neural Networks	Li et al., 2015; Wang et al., 2015, 2016; Zhang et al., 2016; Dong et al., 2017; Li and She, 2017
GF	TF	Karatzoglou et al., 2010; Hidasi and Tikk, 2012; Hidasi, 2015; Kasai and Mishra, 2016; Zhou T. et al., 2017
	FM	Rendle et al., 2011; Cheng et al., 2014; Nguyen et al., 2014; Blondel et al., 2015, 2016; Cao et al., 2016; Juan et al., 2016; Guo et al., 2017; He and Chua, 2017; Lu et al., 2017
HG		Yu et al., 2014; Zheng et al., 2016; Palumbo et al., 2017

### 4.1. Discriminative Matrix Factorization

Intuitively, the goal of an attribute-aware recommender system is to import attributes to improve recommendation performance (either rating prediction or item ranking). In the framework of matrix factorization, an item is rated or ranked according to the latent factors of the item and its corresponding users. In order words, the learning of latent factors in classical matrix factorization depends only on ratings. Thus, the learning may fail due to a lack of training ratings. If we can regularize the latent factors using attributes or make attributes determine how to rate items then matrix factorization methods can be more robust to compensate for the lack of rating information in the training data, especially for those users or items that have very few ratings. In the following, we choose to describe attribute participation from probabilistic perspectives. Learning in Probabilistic Matrix Factorization (PMF) tries to maximize the posterior probability *p*(***W***, ***H*** ∣ ***R***) of two latent factor matrices ***W*** (for users) and ***H*** (for items) given observed entries of training rating matrix ***R***. Clearly, attribute-aware recommender systems claim that we are given an extra attribute matrix ***X***. Then by Bayes' rule, the posterior probability can be shown as follows:

(24) $$\begin{array}{l} \underset{W,H}{\text{argmax }}\underset{\text{Posterior}}{\underset{︸}{p\left(W,H|R,X\right)}}=\underset{W,H}{\text{argmax }}\frac{p\left(R|W,H,X\right)p\left(W,H|X\right)}{p\left(R|X\right)}\\ \;=\underset{W,H}{\text{argmax }}p\left(R|W,H,X\right)p\left(W,H|X\right)\\ \;=\underset{W,H}{\text{argmax }}\underset{\text{Likelihood}}{\underset{︸}{p\left(R|W,H,X\right)}}\underset{\text{Prior}}{\underset{︸}{p\left(W|X\right)p\left(H|X\right)}}. \end{array}$$

**Figure 5:**
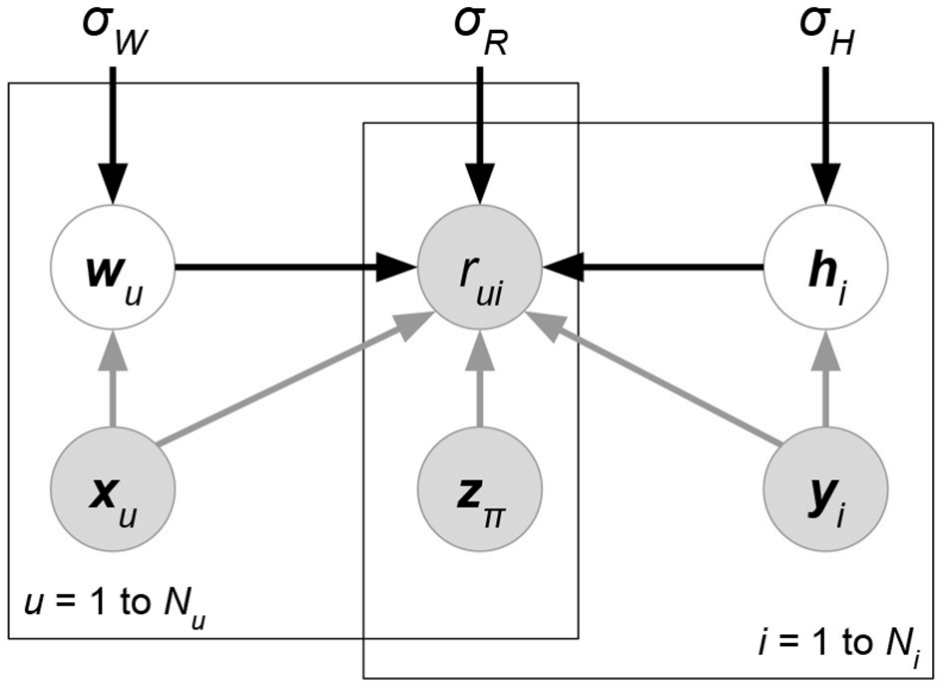
Graphical interpretation of a discriminative probabilistic matrix factorization whose attributes ***X***, ***Y***, ***Z*** are given for ratings and latent factors. User and item-relevant attributes ***X***, ***Y*** could affect the generation of latent factors ***W***, ***H***, or ratings ***R***, while rating-relevant attributes ***Z*** typically determine the rating prediction ***R***. The models of this class may eliminate some of the gray arrows to imply additional independence assumptions between attributes and other factors.

We eliminate the denominator *p*(***R*** ∣ ***X***) since it does not contain variables ***W***, ***H*** for maximization. At the prior part, we follow the independence assumption ***W***⊥***H*** of PMF, though here the independence is given attribute matrix ***X***. Now, compared with classical PMF, both likelihood *p*(***R*** ∣ ***W***, ***H***, ***X***) and prior *p*(***W*** ∣ ***X***)*p*(***H*** ∣ ***X***) could be affected by attributes ***X***. Attributes in the likelihood can directly help predict or rank ratings, while attributes in the priors regularize the learning directions of latent factors. Moreover, some current works assume additional independence between attributes and the matrix factorization formulation. We give graphical interpretation of Discriminative Matrix Factorization in Figure 5. For ease of explanation, we suppose that all the random variables follow a normal distribution $p\left(x\right)=N\left(x|\mu ,\sigma^{2}\right)$ with mean μ and variance σ^2^, or a multivariate normal distribution p(x)=N(x∣μ,Σ) with mean vector ***μ*** and covariance matrix **Σ**. Theoretically, the following models accept other probability distributions.

We further introduce the sub-categories below.

#### 4.1.1. Attributes in a Linear Model

This is the generalized form to utilize attributes in this category. Given the attributes, a weight vector is applied to perform linear regression together with classical matrix factorization ${\text{w}}_{u}^{⊤}{\text{h}}_{i}$. Its characteristics in mathematical form are shown in likelihood functions:

(25) $$\begin{array}{l} \underset{W,H,\theta }{\mathrm{argmax}}\underset{\text{Likelihood}}{\underset{︸}{\prod_{\left(u,i\right)|r_{ui}\in \delta \left(R\right)}N\left(r_{ui}|\mu_{R}=w_{u}^{⊤}h_{i}+\alpha \left(x_{u}\right)+\beta \left(y_{i}\right)+\gamma \left(z_{\pi \left(u,i\right)}\right),\sigma_{R}^{2}\right)}}\\ \;\underset{\text{Prior}}{\underset{︸}{p\left(W|X\right)p\left(H|Y\right)}};, \end{array}$$

where θ = {α, β, γ}, while δ(***R***) denotes the non-missing ratings in the training data, and π(*u, i*) is the column index corresponding to user *u* and item *i*. $\text{X}\in {\mathbb{R}}^{K\times N_{u}},\text{Y}\in {\mathbb{R}}^{K\times N_{i}},\text{Z}\in {\mathbb{R}}^{K\times |\delta \left(\text{R}\right)|}$ denote attribute matrices relevant to user, item, and ratings, respectively, while α, β, γ are their corresponding transformation functions, where attribute space is mapped toward the rating space identical with ${\text{w}}_{u}^{⊤}{\text{h}}_{i}$. Most early models select simple linear transformations, i.e., α(***x***) = ***a***^⊤^***x***, β(***y***) = **b**^⊤^***y***, γ(***z***) = ***c***^⊤^***z***, which has shown a boost in performance, but recent works consider neural networks for non-linear α, β, γ mapping functions. A simple linear regression model can be expressed as a likelihood function of normal distribution $N\left(r|\mu ,\sigma^{2}\right)$ with mean μ and variance σ^2^. Ideally, the distributions of latent factors ***W***, ***H*** will have prior knowledge from attributes ***X***, ***Y***, but we have not yet observed an approach aiming at designing attribute-aware priors as the last two terms of (25).

- **Bayesian Matrix Factorization with Side Information (BMFSI)** (Porteous et al., 2010) is an example in this sub-category. On the basis of Bayesian Probabilistic Matrix Factorization (BPMF) (Salakhutdinov and Mnih, 2008), BMFSI uses a linear combination like (25) to introduce attribute information to rating prediction. It is formulated as:
  (26) $$\begin{array}{l} \underset{W,H,\theta }{\text{argmax }}\underset{\text{Likelihood}}{\underset{︸}{p\left(R|W,H,\theta \right)}}\underset{\text{Priors}}{\underset{︸}{p\left(W\right)p\left(H\right)}}\\ =\underset{W,H,\theta }{\text{argmax}}\underset{\text{Matrix factorization using attributes}}{\underset{︸}{\underset{\left(u,i\right)|r_{ui}\in \delta \left(R\right)}{\prod }N\left(r_{ui}|w_{u}^{⊤}h_{i}+a_{u}^{⊤}x_{u}+b_{i}^{⊤}y_{i},\sigma_{R}^{2}\right)}}\\ \underset{\text{Regularization}}{\underset{︸}{\underset{u}{\prod }N\left(w_{u}|\mu_{u},\Sigma_{u}\right)\underset{i}{\prod }N\left(h_{i}|\mu_{i},\Sigma_{i}\right)}}, \end{array}$$
  where θ = {***a***, ***b***} and δ(***R***) is the set of training ratings. The difference from (25) is that rating attributes ***z*** will be concatenated with either ***x***_*u*_ or ***y***_*u*_, and thus we drop independent weight variable *c* in BMFSI. We ignore other attribute-free designs of BMFSI (e.g., the Dirichlet process).

#### 4.1.2. Attributes in a Bilinear Model

This is a popular method when two kinds of attributes (usually user and item) are provided. Given user attribute matrix ***X*** and item attribute matrix ***Y***, a matrix **A** is used to model the relation between them. The mathematical form can be viewed as follows:

(27) $$\begin{array}{l} \underset{W,H,\theta }{\text{argmax}}\underset{\text{Likelihood}}{\underset{︸}{\underset{\left(u,i\right)|r_{ui}\in \delta \left(R\right)}{\prod }N\left(r_{ui}|\mu_{R}=\alpha \left(x_{u},y_{i}\right)+\beta \left(x_{u}\right)+\gamma \left(y_{i}\right)+b,\sigma_{R}^{2}\right)}}\\ \underset{\text{Prior}}{\underset{︸}{p\left(W|X\right)p\left(H|Y\right)}}, \end{array}$$

where θ = {α, β, γ} are transformation functions from attribute space to rating space. In particular, function α learns the interior dependency between user attributes ***x*** and item attributes ***y***, while β and γ find the extra factors by which ***x*** or ***y*** itself affects the rating result. Compared with (25), the advantage of (27) is that it further considers a set of rating factors that come from the intersections between user and item attributes. However, such a modeling approach cannot work if either user attributes or item attributes are not provided by training data. Prior studies commonly select a simple linear form, called bilinear regression:

(28) $$\begin{array}{l} \mu_{R}=\alpha \left(x_{u},y_{i}\right)+\beta \left(x_{u}\right)+\gamma \left(y_{i}\right)+b\\ \;=x_{u}^{⊤}Ay_{i}+c_{u}^{⊤}x_{u}+d_{i}^{⊤}y_{i}+b\\ \;={\tilde{x}}_{u}^{⊤}\tilde{A}{\tilde{y}}_{i}. \end{array}$$

In fact, as mentioned in Lu et al. (2016), ${\text{c}}_{u}^{⊤}{\text{x}}_{u}+{\text{d}}_{i}^{⊤}{\text{y}}_{i}+\text{b}$ can be absorbed into ${\text{x}}_{u}^{⊤}\text{A}{\text{y}}_{i}$ and written in the form ${\tilde{x}}_{u}^{⊤}\tilde{A}{\tilde{y}}_{i}$ by appending a new dimension whose value is fixed to 1 for each ***x*** and ***y***.

Works in this category differ on whether the bilinear term is explicit or implicit. Also, the latent factor matrices ***W***, ***H*** are inherently included in the bilinear form. Specifically, (28) implies that the form of the dot product of two linear-transformed attributes ***w***_*u*_ = ***Sx***_*u*_ and ***h***_*i*_ = ***Ty***_*i*_ since it can be reformed as ${\text{w}}_{u}^{⊤}{\text{h}}_{i}={\text{x}}_{u}^{⊤}\left({\text{S}}^{⊤}\text{T}\right){\text{y}}_{i}$ where ***A*** = ***S***^⊤^***T***. Some works such as the Regression-based Latent Factor Model (see below) choose to softly constrain ***w***_*u*_ ≈ ***Sx***_*u*_ and ***h***_*i*_ ≈ ***Ty***_*i*_ using priors *p*(***W*** ∣ ***X***) and, *p*(***H*** ∣ ***Y***).

**Figure 6:**
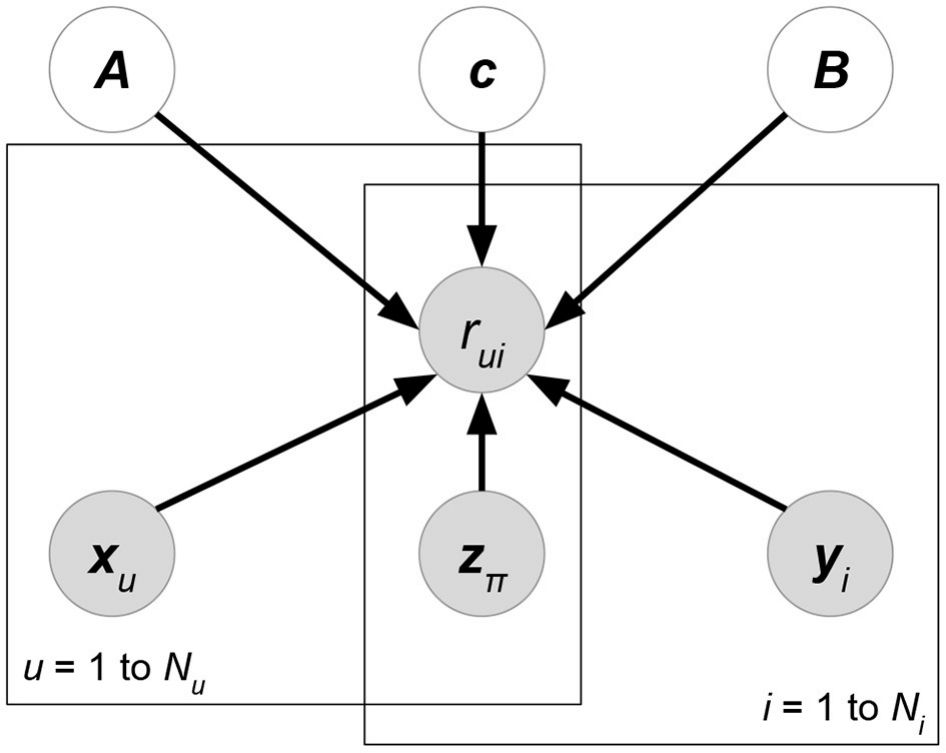
Matchbox.

**Figure 7:**
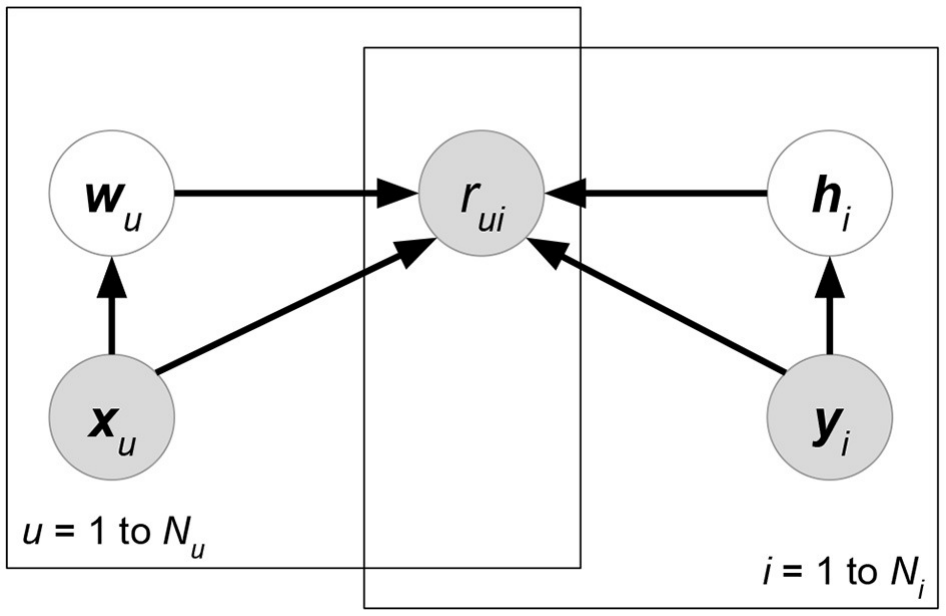
KPMF.

**Figure 8:**
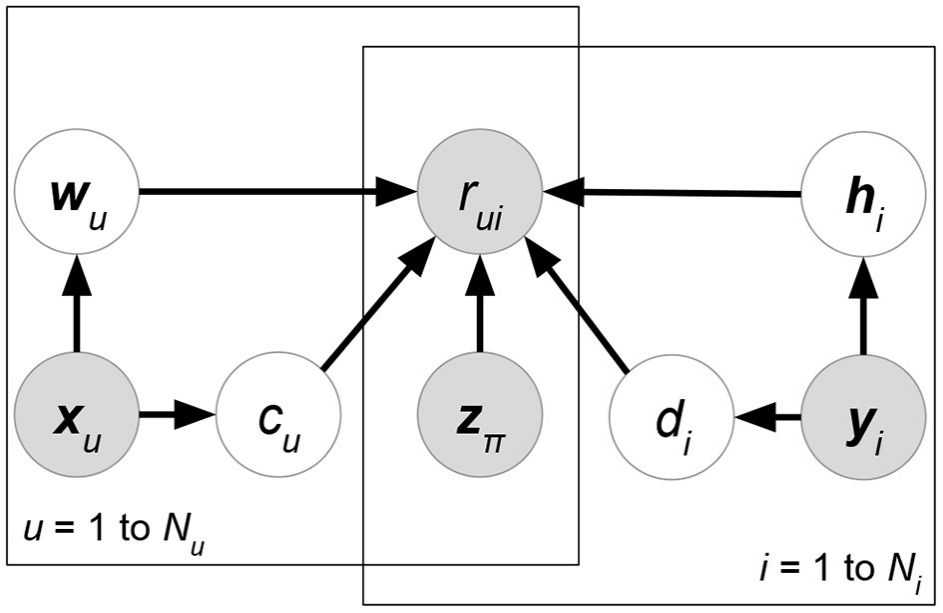
RLFM.

- **Matchbox** (Stern et al., 2009) (Figure 6). Let ***X***, ***Y***, ***Z*** be the attribute matrices with respect to users, items, and ratings, respectively. Matchbox assumes a rating is predicted by the linear combinations of ***X***, ***Y***, ***Z***:
  (29) $$\begin{array}{l} \underset{A,B,c}{\text{argmax }}\underset{\text{Likelihood}}{\underset{︸}{p\left(R|A,B,c,X,Y,Z\right)}}\underset{\text{Prior}}{\underset{︸}{p\left(c\right)p\left(A\right)p\left(B\right)}}\\ \;=\underset{A,B,c}{\text{argmax}}\underset{\text{Matrix factorization using attributes}}{\underset{︸}{\underset{\left(u,i\right)|r_{ui}\in \delta \left(R\right)}{\prod }N\left(r_{ui}|x_{u}^{⊤}A^{⊤}By_{i}+c^{⊤}z_{\pi \left(u,i\right)},\sigma_{R}^{2}\right)}}\\ \underset{\text{Regularization}}{\underset{︸}{\begin{array}{c} \;\prod_{m}N\left(c_{m}|\mu_{cm},\sigma_{cm}^{2}\right)\prod_{\left(u,k\right)}N\left(a_{uk}|\mu_{Auk},\sigma_{Auk}^{2}\right)\prod_{\left(i,l\right)}N\left(b_{il}|\mu_{Bil},\sigma_{Bil}^{2}\right) \end{array}}} \end{array}$$
  where δ(***R***) is the set of non-missing entries in rating matrix *R*. ***x***_*u*_, ***y***_*i*_ represents the attribute set of user *u* or item *i*. ***z***_(*u,i*)_ denotes the rating-relevant attributes associated with user *u* and item *i*. Note that (29) defines latent factors ***W*** = ***AX***, ***H*** = ***BY***, and then we just have to learn the shared weight matrices ***A***, **B**. The prior distributions of ***A***, **B**, ***c*** are further factorized, which assumes that all the weight entries in these matrices are independent of each other.
- **Friendship-Interest Propagation (FIP)** (Yang et al., 2011) (Figure 7). Following the notations from the previous RLFM introduction, FIP considers two types of attribute matrices: ***X*** and ***Y***. Based on vanilla matrix factorization, FIP encodes attribute information by modeling the potential correlations between ***X*** and ***Y***:
  (30) $$\begin{array}{l} \underset{W,H,A,B,C}{\text{argmax}}\underset{\text{Likelihood}}{\underset{︸}{p\left(R|W,H,C,X,Y\right)}}\underset{\text{Prior}}{\underset{︸}{p\left(W|A,X\right)p\left(H|B,Y\right)}}\\ \;=\underset{W,H,A,B,C}{\text{argmax}}\underset{\text{Matrix factorization using attributes}}{\underset{︸}{\underset{\left(u,i\right)|r_{ui}\in \delta \left(R\right)}{\prod }N\left(r_{ui}|w_{u}^{⊤}h_{i}+x_{u}^{⊤}Cy_{i},\sigma_{R}^{2}\right)}}\\ \;\underset{\text{Regularization using attributes}}{\underset{︸}{\underset{u}{\prod }N\left(w_{u}|Ax_{u},\Sigma_{W}\right)\underset{i}{\prod }N\left(h_{i}|By_{i},\Sigma_{H}\right)}} \end{array}$$
  where matrix ***C*** forms the correlations between attribute matrices ***X*** and ***Y***.
- **Regression-based Latent Factor Model (RLFM)** (Agarwal and Chen, 2009) (Figure 8). Given three types of attribute matrices: user-relevant ***X***, item-relevant ***Y***, and rating-relevant ***Z***, RLFM models them in different parts of biased matrix factorization. ***X***, ***Y*** serve as the hyperparameters of latent factors, while ***Z*** joins the regression framework to predict ratings together with latent factors. RLFM can be written as:
  (31) $$\begin{array}{l} \underset{W,H,\theta }{\text{argmax }}\underset{\text{Likelihood}}{\underset{︸}{p\left(R|W,H,c,d,\gamma ,Z\right)}}\\ \underset{\text{Prior}}{\underset{︸}{p\left(W|A,X\right)p\left(H|B,Y\right)p\left(c|\alpha ,X\right)p\left(d|\beta ,Y\right)}}\\ =\underset{W,H,\theta }{\text{argmax}}\underset{\text{Matrix factorization using attributes}}{\underset{︸}{\underset{\left(u,i\right)|r_{ui}\in \delta \left(R\right)}{\prod }N\left(r_{ui}|w_{u}^{⊤}h_{i}+c_{u}+d_{i}+\gamma^{⊤}z_{\pi \left(u,i\right)},\sigma_{R}^{2}\right)}}\\ \underset{\text{Regularization using attributes}}{\underset{︸}{\begin{array}{c} \prod_{u}N\left(w_{u}|Ax_{u},\Sigma_{W}\right)N\left(c_{u}|\alpha^{⊤}x_{u},\sigma_{c}^{2}\right)\prod_{i}N\left(h_{i}|By_{i},\Sigma_{H}\right)N\left(d_{i}|\beta^{⊤}y_{i},\sigma_{d}^{2}\right) \end{array}}} \end{array}$$
  where θ = {***c***, ***d***, ***A***, ***B***, ***α***, ***β***, ***γ***} and δ(***R***) is the set of non-missing ratings for training. Biased matrix factorization adds two vectors ***c***, ***d*** to learn the biases for each user or item. Parameters ***A***, ***B***, ***α***, ***β***, **γ** map attributes with latent factors (for ***X***, ***Y***) or rating prediction (for ***Z***).

#### 4.1.3. Attributes in a Similarity Matrix

In this case, a similarity matrix that measures the closeness of attributes between users or between items is presented. Given the user attribute matrix $\text{X}\in {\mathbb{R}}^{D\times N_{u}}$, where *N*_*u*_ is the number of users and *D* is the user attribute dimension, a similarity matrix $\text{S}\in {\mathbb{R}}^{N_{u}\times N_{u}}$ is computed. There are many metrics for similarity calculation, such as Euclidean distance and kernel functions. The similarity matrix is then used for matrix factorization or other solutions. The special quality of this case is that human knowledge is involved in determining how the interactions between attributes should be modeled. One example is Kernelized Probabilistic Matrix Factorization, which utilizes both a user similarity matrix and item a similarity matrix.

**Figure 9:**
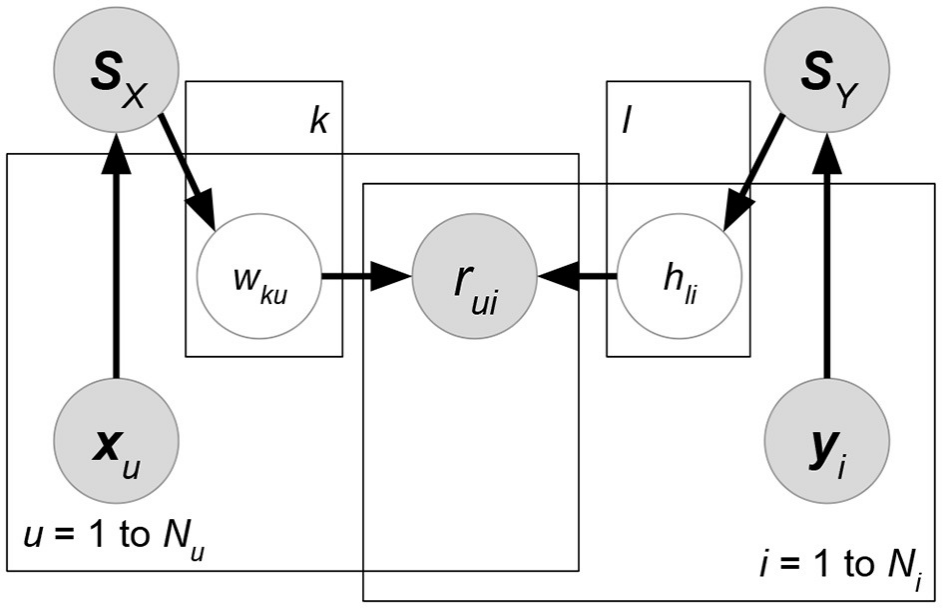
FIP.

- **Kernelized Probabilistic Matrix Factorization (KPMF)** (Zhou et al., 2012) (Figure 9). Let *K, N*_*u*_, *N*_*i*_ be the number of latent factors, users, and items. Given user-relevant attribute matrix $\text{X}\in {\mathbb{R}}^{K\times N_{u}}$ or item-relevant attribute matrix $\text{Y}\in {\mathbb{R}}^{K\times N_{i}}$, we can always obtain a similarity matrix ${\text{S}}_{\text{X}}\in {\mathbb{R}}^{N_{u}\times N_{u}}$ or ${\text{S}}_{\text{Y}}\in {\mathbb{R}}^{N_{i}\times N_{i}}$ where each entry stores a pre-defined similarity between a pair of users or items. KPMF then formulates the similarity matrix as the prior of its corresponding latent factor matrix:
  (32) $$\begin{array}{l} \underset{W,H}{\text{argmax }}\underset{\text{Likelihood}}{\underset{︸}{p\left(R|W,H\right)}}\underset{\text{Prior}}{\underset{︸}{p\left(W|X\right)p\left(H|Y\right)}}\\ =\underset{W,H}{\text{argmax}}\underset{\text{Matrix factorization}}{\underset{︸}{\underset{\left(u,i\right)|r_{ui}\in \delta \left(R\right)}{\prod }N\left(r_{ui}|w_{u}^{⊤}h_{i},\sigma_{R}^{2}\right)}}\\ \underset{\text{Regularization using attributes}}{\underset{︸}{\underset{k}{\prod }N\left(w^{k}|0,S_{X}\right)\underset{l}{\prod }N\left(h^{l}|0,S_{Y}\right).}} \end{array}$$
  Here, we use subscripts ***w***_*u*_ to denote the *u*-th column vector of a matrix ***W***, while superscripts ***w***^*k*^ imply the *k*-th row vector of ***W***. Intuitively, the similarity matrices control the learning preferences of user or item latent factors. If two users have similar user-relevant attributes (i.e., they have a higher similarity measure in ***S***_***X***_), then their latent factors are forced to be closer during the matrix factorization learning.

Neural Collaborative Filtering (He et al., 2017) (NCF) is a model that combines a linear structure and a neural network. He et al. (2018) further improves it by using CNN on top of the outer product of user and item embeddings. However, the authors only used user and item one-hot encoding vectors as their input. Though they mentioned that it could be easily modified to accommodate additional attributes, it was not clearly demonstrated. Therefore, we do not include it in either of the categories since it is beyond the scope of our survey (though it is similar to Discriminative Matrix Factorization). We still include NCF as a baseline model in the empirical comparison section, as well as including a slight modification of the model that takes additional attributes as inputs as a competitor. A brief introduction of the model and its variance will be given in section 5.1.1.

### 4.2. Generative Matrix Factorization

**Figure 10:**
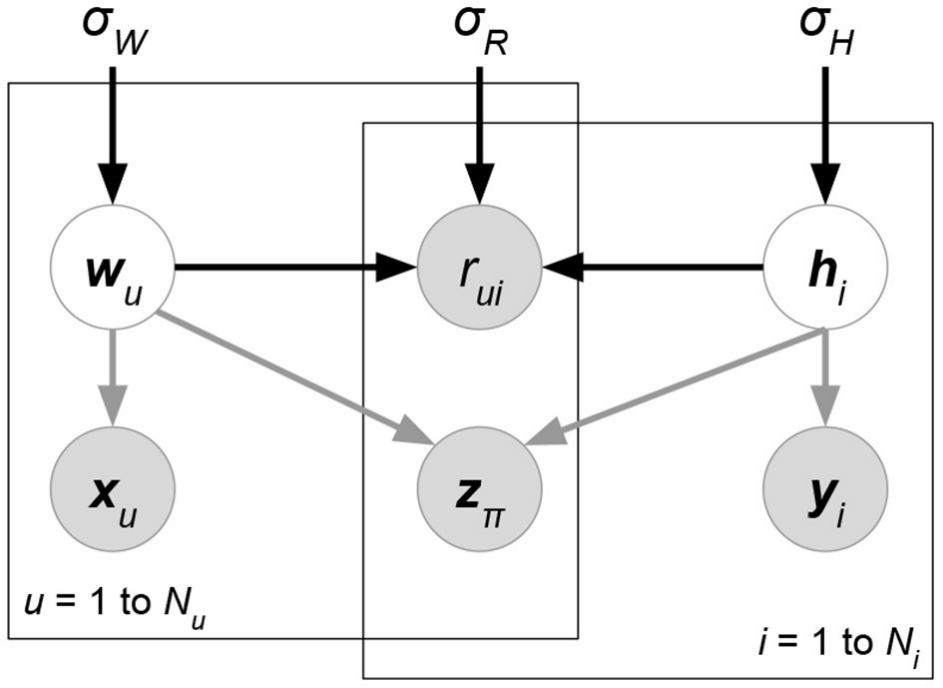
Graphical interpretation of generative probabilistic matrix factorization, whose attributes ***X***, ***Y***, ***Z***, together with ratings, are generated or predicted by latent factors. User and item-relevant attributes ***X***, ***Y*** could be generated by corresponding latent factors ***W***, ***H***, respectively. Rating-relevant attributes ***Z*** are likely to result from both ***W*** and ***H***. For models of this class, some of the gray arrows are removed to represent their additional independence assumptions about attribute generation.

In Probabilistic Matrix Factorization (PMF), ratings are generated by the interactions of user or item latent factors. However, the PMF latent factors are not limited to rating generation. We can also generate attributes from latent factors. Mathematically, using Bayes' rule, we maximize a posteriori as follows:

(33) $$\begin{array}{l} \underset{W,H}{\text{argmax }}\underset{\text{Posterior}}{\underset{︸}{p\left(W,H|R,X\right)}}=\underset{W,H}{\text{argmax}}\frac{p\left(R,X|W,H\right)p\left(W,H\right)}{p\left(R,X\right)}\\ \;=\underset{W,H}{\text{argmax }}p\left(R,X|W,H\right)p\left(W,H\right)\\ \;=\underset{W,H}{\text{argmax }}\underset{\text{Likelihood}}{\underset{︸}{p\left(R|W,H\right)p\left(X|W,H\right)}}\\ \;\underset{\text{Prior}}{\underset{︸}{p\left(W\right)p\left(H\right)}}. \end{array}$$

where *p*(***R***, ***X***) does not affect the posterior maximization. We again assume independence ***R***⊥***X*** given latent factors ***W***, ***H*** in (33), which is commonly adopted in related work. Furthermore, ***X*** may share either latent factors ***W*** [i.e., *p*(***X*** ∣ ***W***)] or ***H*** [i.e., *p*(***X*** ∣ ***H***)] with ***R*** but not both due to matrix factorization having more generalization capability. We give the graphical interpretation of Generative Matrix Factorization in Figure 10.

The following relevant works are classified in this category. For simplicity, all the probabilities follow a normal distribution, i.e, $p\left(x\right)=N\left(x|\mu ,\sigma^{2}\right)$ (i.e., squared loss objective) with mean μ and variance σ^2^ (or mean vector **μ** and covariance matrix **Σ** for multivariate normal distributions). However, the example models are never restricted to within normal distributions.

There are two different branches in this direction. On the one hand, earlier works again use the matrix factorization technique to generate attributes from user or item latent factors. This can be seen as a linear mapping between latent factors and attributes. On the other hand, with the help of deep neural networks, recent works combine matrix factorization and deep autoencoders to realize non-linear mappings for attribute generation. We will introduce them in the following sections.

#### 4.2.1. Attributes in Multiple Matrix Factorization

Similar to PMF ***R*** ≈ ***W***^⊤^***H*** for rating distributions; attributes distributions are modeled using another matrix factorization form. Given user attribute matrix ***X***, item attribute matrix ***Y***, and rating attribute matrix ***Z***, they can be factorized as ***X*** ≈ ***A***^⊤^***W***, ***Y*** ≈ **B**^⊤^***H*** of, low rank. Specifically, its objective function is written as:

(34) $$\begin{array}{l} \underset{W,H,A,B}{\text{argmax}}\\ \underset{\text{Likelihood}}{\underset{︸}{\begin{array}{c} \prod_{\left(u,i\right)|r_{ui}\in \delta \left(R\right)}N\left(r_{ui}|\mu_{R}=w_{u}^{⊤}h_{i},\sigma_{R}^{2}\right)\prod_{\left(j,u\right)}N\left(x_{ju}|a_{j}^{⊤}w_{u},\sigma_{X}^{2}\right)\prod_{\left(v,i\right)}N\left(y_{vi}|b_{v}^{⊤}h_{i},\sigma_{Y}^{2}\right) \end{array}}}\\ \underset{\text{Likelihood}}{\underset{︸}{\underset{\left(u,i\right)|r_{ui}\in \delta \left(R\right)}{\prod }N\left(z_{ui}|w_{u}^{⊤}Ch_{i},\sigma_{Z}^{2}\right)}}\underset{\text{Prior}}{\underset{︸}{p\left(W\right)p\left(H\right)}}, \end{array}$$

where δ(***R***) denotes the non-missing entries of matrix ***R***. The insight of (34) is to share the latent factors ***W***, ***H*** in multiple factorization tasks. ***W*** is shared with user attributes, while ***H*** is shared with item attributes. ***Z*** requires the sharing of both ***W*** and ***H*** due to user- and item-specific rating attributes. Therefore, the side information of ***X***, ***Y***, and ***Z*** can indirectly transfer to rating prediction. Auxiliary matrices ***A***, **B**, and ***C*** learn the mappings between latent factors and attributes. With respect to the mathematical form of matrix factorization, the expectation of feature values is linearly correlated with its corresponding latent factors.

**Figure 11:**
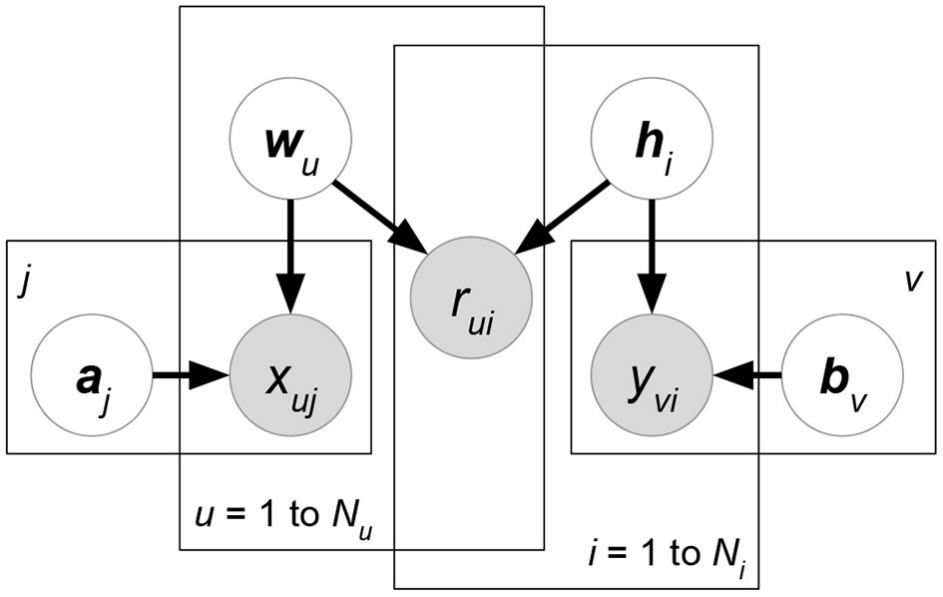
CMF.

- **Collective Matrix Factorization (CMF)** (Singh and Gordon, 2008) (Figure 11). Here, we introduce a common model in this sub-category. The CMF framework relies on the combination of multiple matrix factorization objective functions. CMF first builds the MF for rating matrix ***R***. User- and item-relevant attribute matrices ***X***, ***Y*** are then appended to the matrix factorization objectives. Overall, we have:
  (35) $$\begin{array}{l} \underset{W,H,A,B}{\text{argmax }}\underset{\text{Likelihood}}{\underset{︸}{p\left(R|W,H\right)p\left(X|W,A\right)p\left(Y|H,B\right)}}\underset{\text{Prior}}{\underset{︸}{p\left(W\right)p\left(H\right)p\left(A\right)p\left(B\right)}}\\ =\underset{W,H,A,B}{\text{argmax}}\underset{\text{Matrix factorization of }R}{\underset{︸}{\underset{\left(u,i\right)|r_{ui}\in \delta \left(R\right)}{\prod }N\left(r_{ui}|w_{u}^{⊤}h_{i},\sigma_{R}^{2}\right)}}\underset{\text{Matrix factorization of }X}{\underset{︸}{\underset{\left(j,u\right)}{\prod }N\left(x_{ju}|a_{j}^{⊤}w_{u},\sigma_{X}^{2}\right)}}\\ \underset{\text{Matrix factorization of }Y}{\underset{︸}{\underset{\left(v,i\right)}{\prod }N\left(y_{vi}|b_{v}^{⊤}h_{i},\sigma_{Y}^{2}\right)}}\\ \underset{\text{Regularization}}{\underset{︸}{\begin{array}{c} \prod_{u}N\left(w_{u}|0,\Sigma_{W}\right)\prod_{i}N\left(h_{i}|0,\Sigma_{H}\right)\prod_{j}N\left(a_{j}|0,\Sigma_{A}\right)\prod_{v}N\left(b_{v}|0,\Sigma_{B}\right) \end{array}}} \end{array}$$
  where δ(***R***), δ(***X***), δ(***Y***) denote the non-missing entries of matrix ***R***, ***X***, ***Y*** that are generated by latent factor matrices ***W***, ***H***, ***A***, **B** of zero-mean normal priors (i.e., *l*2 regularization). In (35), ***W***, ***H*** are shared by at least two matrix factorization objectives. Attribute information in ***X***, ***Y*** is transferred to rating prediction ***R*** through sharing the same latent factors. Note that CMF is not limited to three matrix factorization objectives (35).

#### 4.2.2. Attributes in Deep Neural Networks

In deep neural networks, an autoencoder is usually used to learn the latent representation of observed data. Specifically, the model tries to construct an encoder E and a decoder D, where the encoder learns to map from possibly modified attributes $\tilde{X}$ to low-dimensional latent factors, and the decoder recovers original attributes ***X*** from latent factors. Moreover, activation functions in autoencoders can reflect non-linear mappings between latent factors and attributes, which may capture the characteristics of attributes more accurately.

To implement an autoencoder, we first generate another attribute matrix $\tilde{X}$ from ***X***. $\tilde{X}$ could be the same as ***X*** or could be different due to corruption, e.g., adding random noise. Autoencoders aim to predict the original ***X*** using latent factors that are inferred from generated $\tilde{X}$. Here, attributes serve not only as the generation results ***X*** but also as the prior knowledge $\tilde{X}$ of latent factors. Let us review Bayes' Rule to figure out where autoencoders appear for generative matrix factorization:

(36) $$\begin{array}{l} \underset{W,H}{\text{argmax }}p\left(W,H|R,X,\tilde{X}\right)=\underset{W,H}{\text{argmax}}\frac{p\left(R,X|W,H,\tilde{X}\right)p\left(W,H|\tilde{X}\right)}{p\left(R,X|\tilde{X}\right)}\\ \;=\underset{W,H}{\text{argmax }}p\left(R,X|W,H,\tilde{X}\right)p\left(W,H|\tilde{X}\right)\\ =\underset{W,H}{\text{argmax }}\underset{\text{Likelihood}}{\underset{︸}{\underset{\text{Matrix factorization}}{\underset{︸}{p\left(R|W,H,\tilde{X}\right)}}\underset{\text{Decoder}D}{\underset{︸}{p\left(X|W,H,\tilde{X}\right)}}}}\underset{\text{Prior with assumption }W⊥H|\tilde{X}}{\underset{︸}{\underset{\text{Encoder}E}{\underset{︸}{p\left(W|\tilde{X}\right)p\left(H|\tilde{X}\right)}}}}. \end{array}$$

*p*(***R***, ***Y*** ∣ ***Ỹ***) is eliminated due to irrelevance in maximization of (36). By sharing latent factors ***W***, ***H*** between autoencoders and matrix factorization, attribute information can affect the learning of rating prediction. Modeling D with normal distributions, we can conclude that the expectation of attributes ***X*** is non-linearly mapped from latent factors ***W***, ***H***. Although latent factors have priors from attributes, we categorize relevant works as generative matrix factorization, since we explicitly model attribute distributions in the decoder part of autoencoders.

**Figure 12:**
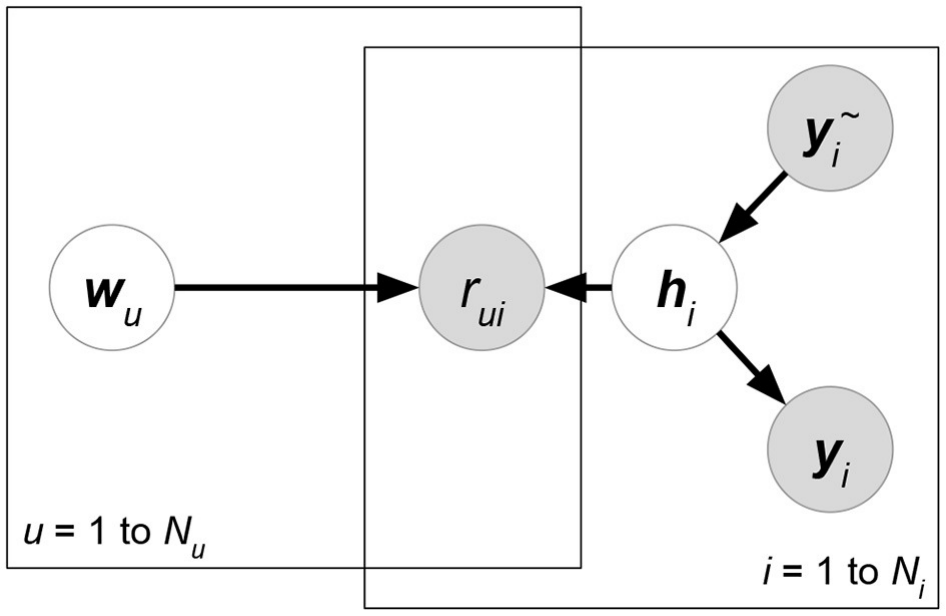
CDL.

- **Collaborative Deep Learning (CDL)** (Wang et al., 2015) (Figure 12). This model uses a combination of collaborative filtering and Stacked Denoising Auto-Encoder (SDAE). Since the model claims to exploit item attributes ***Y*** only, in the following introduction we define $Y=X,\tilde{Y}=\tilde{X}$ in (36). In SDAE, input attribute ***Ỹ*** is not equivalent to ***Y*** due to the addition of random noise to ***Ỹ***. CDL implicitly adds several independence assumptions (***R***⊥***Ỹ*** ∣ ***W***, ***H***), (***Y***⊥***W*** ∣ ***H***, ***Ỹ***), (***W***⊥***Ỹ***) to formulate its model. Then, using identical notations in CMF introduction, normal distributions N are again applied to CDL:
  (37) $$\begin{array}{l} \underset{W,H,\theta ,\phi }{\text{argmax }}\underset{\text{Likelihood}}{\underset{︸}{p\left(R,|W,H\right)p\left(Y|H,\tilde{Y}\right)}}\underset{\text{Prior}}{\underset{︸}{p\left(H|\tilde{Y}\right)p\left(W\right)}}\\ =\underset{W,H,\theta ,\phi }{\text{argmax}}\underset{\text{Matrix factorization}}{\underset{︸}{\underset{\left(u,i\right)|r_{ui}\in \delta \left(R\right)}{\prod }N\left(r_{ui}|w_{u}^{⊤}h_{i},\sigma_{R}^{2}\right)}}\\ \underset{\text{Stacked denoising auto-encoder for }Y}{\underset{︸}{\underset{i}{\prod }N\left(y_{i}|D_{\phi }\left(E_{\theta }\left({\tilde{y}}_{i}\right)\right),\Sigma_{Y}\right)N\left(h_{i}|E_{\theta }\left({\tilde{y}}_{i}\right),\Sigma_{H}\right)}}\\ \underset{\text{Regularization}}{\underset{︸}{\underset{u}{\prod }N\left(w_{u}|0,\Sigma_{W}\right)}}. \end{array}$$
  Functions E,D indicate the encoder and the decoder of SDAE. The two functions could be formed by multi-layer perceptrons whose parameters are denoted by ***θ***, ***ϕ***. The distribution of attribute matrix ***Y*** to be modeled in the decoder part can be clearly seen. Last but not least, the analysis from (36) to (37) implies that other ideas, user-relevant attributes for example, could be involved naturally in CDL, as long as we remove more independence assumptions.

### 4.3. Generalized Factorization

**Figure 13:**
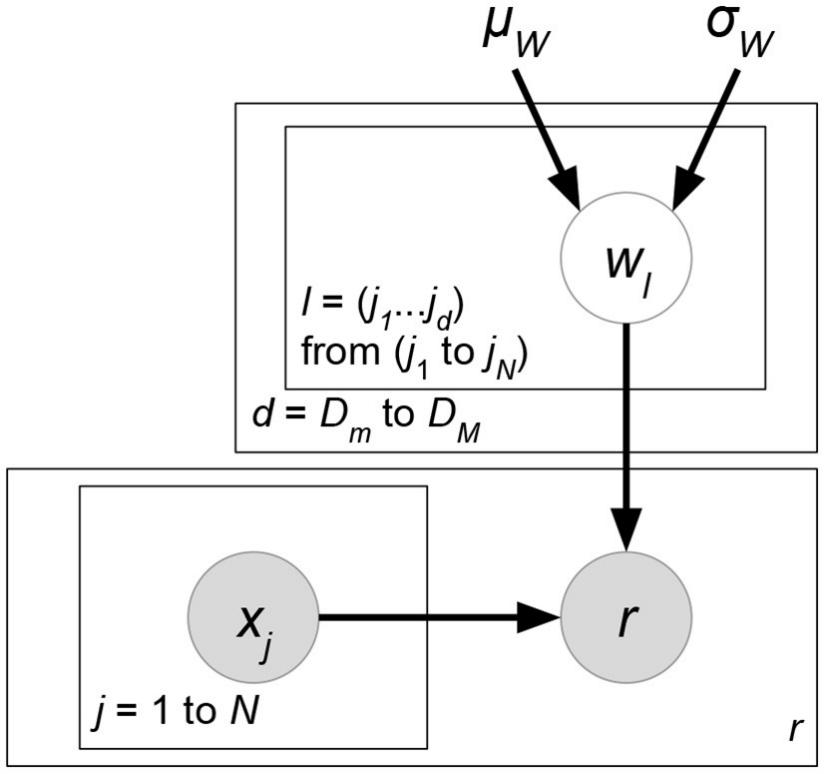
*w*-weighted generalization.

**Figure 14:**
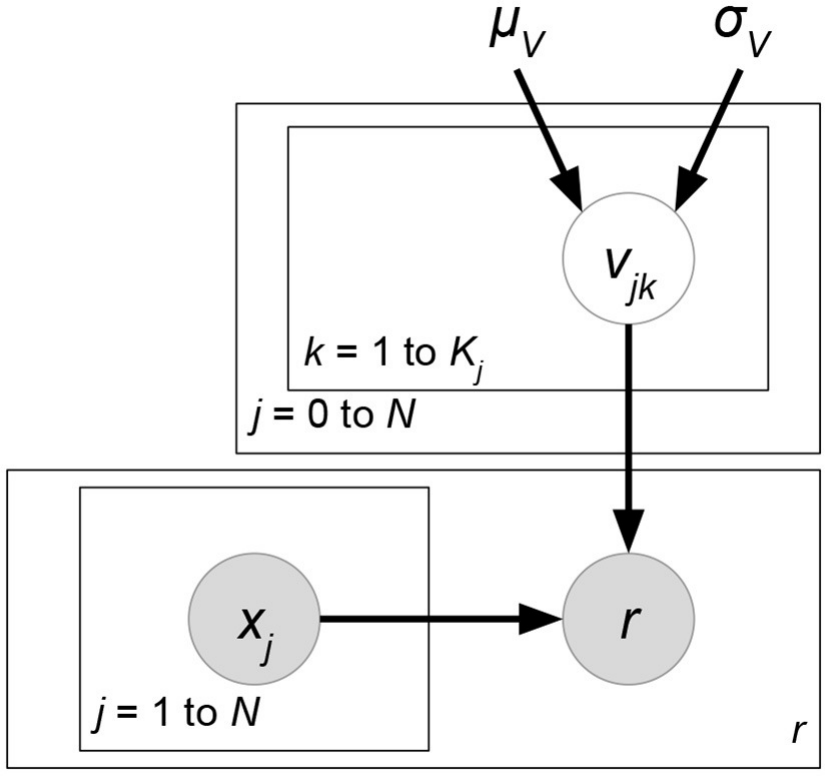
*v*-approximate generalization.

**Figure 15:**
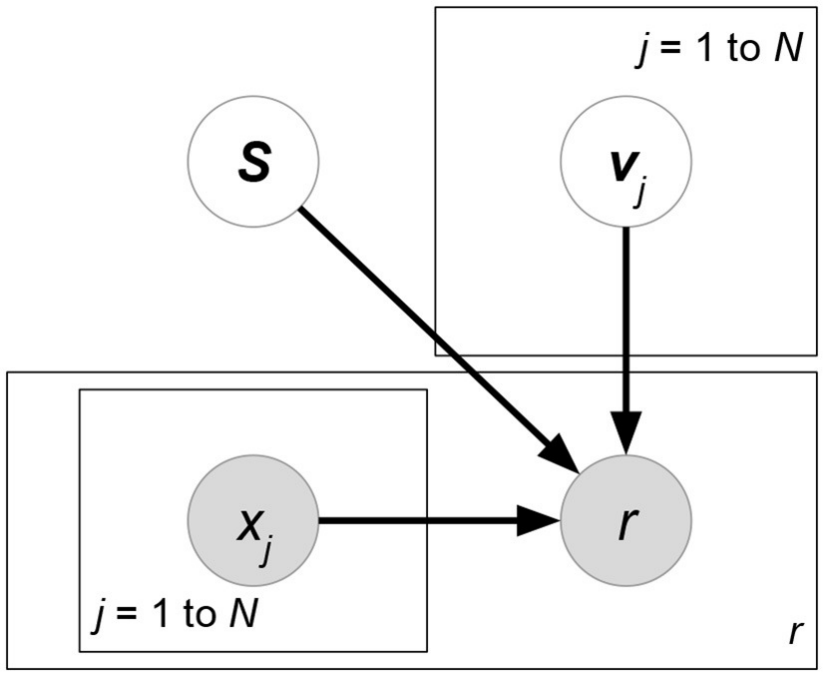
TF.

**Figure 16:**
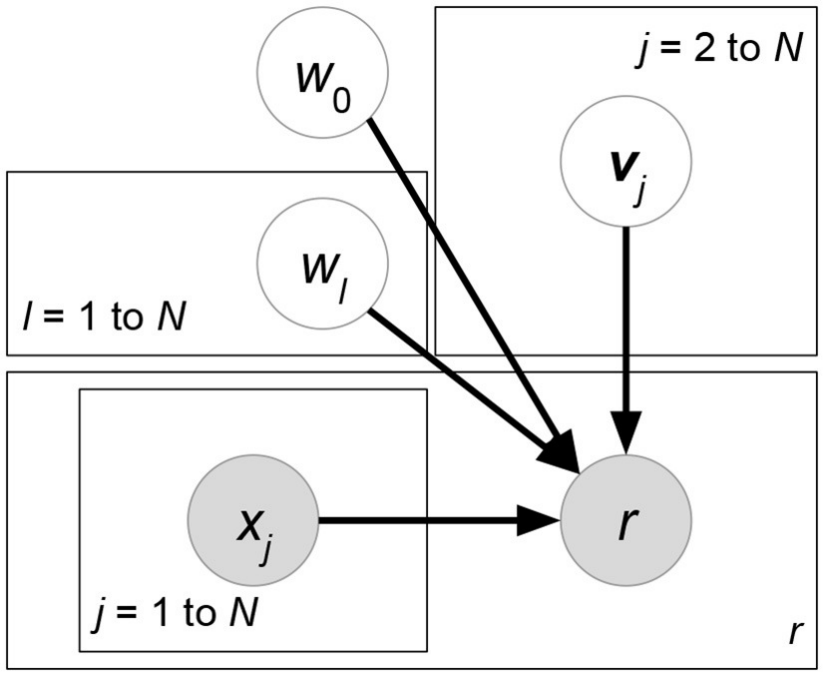
FM.

Thanks to the success of matrix factorization in recommender systems, advanced works have emerged on generalizing the concept of matrix factorization in order to extract more information from attributes or interactions between users and items. The works classified in either sections 4.1 or 4.2 propose to design attribute-aware components on the basis of PMF. They explicitly express an assumption of vanilla PMF: the existence of a latent factor matrix ***W*** to represent user preferences and another matrix ***H*** for items. However, the works classified in this section do not regard ***W*** and ***H*** as special features of the models. Rather, such works propose an expanded latent factor space shared by users, items, and attributes. Here, neither users nor items are special entities in a recommender system. They are simply considered as categorical attributes. Taking rating *r*_*ui*_ as an example, it implies that we have a one-hot user-encoding vector where all the entries are 0 except for the *u*-th entry; similarly, we also have a one-hot item-encoding vector of the *i*-th entry, 1. Thus, external attributes ***X*** can be involved simply in the matrix-factorization-based models, because now users and items are also attributes whose interactions commonly predict or rank ratings. We first propose the most generalized version of interpretation: Given a rating *r* and its corresponding attribute vector ***x*** ∈ ℝ^*N*^, then we make rating estimate (Figure 13):

(38) $$\begin{array}{l} \underset{w}{\text{argmax}}\prod_{r\in \delta (R)}N(r|\mu_{R}\\ =\sum_{d=D_{m}}^{D_{M}}\sum_{j_{1}=1}^{N}\sum_{j_{2}=j_{1}+1}^{N}\text{​}\ldots \text{​}\sum_{j_{d}=j_{d-1}+1}^{N}w_{j_{1}j_{2}\ldots j_{d}}\left(x_{j_{1}}x_{j_{2}}\ldots x_{j_{d}}\right),\sigma_{R}^{2}), \end{array}$$

where δ(***R***) indicates the set of observed ratings in training data. Variable *d* ∈ {0} ∪ ℕ determines the *d*th-order multiplication interaction between attributes *x*_*j*_. As *d* = 0, we introduce an extra bias weight *w*_0_ ∈ ℝ in (38). The large number of parameters *w* ∈ ℝ is very likely to overfit training ratings due to the dimensionality curse. To alleviate overfitting problems, the ideas in matrix factorization are applied here. For higher values of *d*, it is assumed that each *w* is a function of low-dimensional latent factors:

(39) $$\begin{array}{l} w_{j_{1}j_{2}\ldots j_{d}}=f_{d}\left(v_{j_{1}},v_{j_{2}},\ldots ,v_{j_{d}}\right), \end{array}$$

where ${\text{v}}_{j}\in {\mathbb{R}}^{K_{j}}$ implies the *K*-dimensional (*K*_*j*_ ≪ *N*∀*j*) latent factor or representation vector for each element *x*_*j*_ of ***x*** (Figure 14). Function *f*_*d*_ maps these *d* vectors to a real-valued weight. Our learning parameters then become ***v***. The overall number of parameters (*D*_*m*_ ≤ *d* ≤ *D*_*M*_) decreases from $\overset{D_{M}}{\underset{d=D_{m}}{\sum }}\frac{n!}{d!\left(n-d\right)!}=O\left(2^{N}\right)$ to $\overset{N}{\underset{j=1}{\sum }}K_{j}=O\left(NK\right)$ where $K={\text{max}}_{1\leq j\leq N}K_{j}$. Next, we prove that matrix factorization is a special case of (38). Let *D*_*m*_ = *D*_*M*_ = 2 and ***x*** be the concatenation of one-hot encoding vectors of users as well as items. Also, we define $f_{2}\left(\text{v},\text{y}\right)={\text{v}}^{⊤}\text{y}$. Then, for rating *r*_*ui*_ of user *u* to item *i*, we have:

(40) $$\begin{array}{l} \underset{v}{\text{argmax}}\underset{r_{ui}\in \delta \left(R\right)}{\prod }N\left({\hat{r}}_{ui}|\mu_{R}=\overset{N}{\underset{j_{1}=1}{\sum }}\overset{N}{\underset{j_{2}=j_{1}+1}{\sum }}v_{j_{1}}^{⊤}v_{j_{2}}\left(x_{j_{1}}x_{j_{2}}\right)=v_{u}^{⊤}v_{N_{u}+i},\sigma_{R}^{2}\right), \end{array}$$

where *N*_*u*_ denotes the number of users. Equation (40) is essentially equivalent to matrix factorization. In this class, the existing works either generalize or improve two early published models: Tensor Factorization (TF) (Figure 15) and the Factorization Machine (FM) (Figure 16). Both models can be viewed as special cases of (38). We introduce TF and FM in the sections below.

#### 4.3.1. TF-Extended Models

Tensor Factorization (TF) (Karatzoglou et al., 2010) requires the input features to be categorical. Attribute vector ***x*** ∈ {0, 1}^*N*^ is the concatenation of *D* one-hot encoding vectors. (*D* − 2) categorical rating-relevant attributes form their own binary one-hot representations. The additional two one-hot vectors represent IDs of users and items, respectively. As a special case of (38), TF fixes *D*_*m*_ = *D*_*M*_ = *D* to build a single *D*-order interaction between attributes. Since weight function *f*_*D*_ in (39) allows individual dimensions *K*_*j*_ for each latent factor vector ***v***_*j*_, TF defines a tensor $S\in {\mathbb{R}}^{K_{1}\times K_{2}\times \ldots \times K_{D}}$ to exploit the tensor product of all latent factor vectors. In sum, (38) is simplified as follows:

(41) $$\begin{array}{l} \mu_{R}=\overset{N}{\underset{j_{1}=1}{\sum }}\overset{N}{\underset{j_{2}=j_{1}+1}{\sum }}\ldots \overset{N}{\underset{j_{D}=j_{D-1}+1}{\sum }}f_{D}\left(v_{j_{1}},v_{j_{2}},\ldots ,v_{j_{D}}\right)\left(x_{j_{1}}x_{j_{2}}\ldots x_{j_{D}}\right)\\ \;=f_{D}\left(v_{l_{1}},v_{l_{1}},\ldots ,v_{l_{D}}\right)\text{ as }x_{l_{1}}=x_{l_{2}}=\ldots \\ \;=x_{l_{D}}=1,\text{other }x=0\\ \;=\langle S,v_{l_{1}},v_{l_{2}},\ldots ,v_{l_{D}}\rangle \\ \;=\overset{K_{1}}{\underset{k_{1}=1}{\sum }}\overset{K_{2}}{\underset{k_{2}=1}{\sum }}\ldots \overset{K_{D}}{\underset{k_{D}=1}{\sum }}s_{k_{1}k_{2}\ldots k_{D}}v_{l_{1}k_{1}}v_{l_{2}k_{2}}\ldots v_{l_{D}k_{D}} \end{array}$$

where function *f*(·) = < · > denotes the tensor product. Note that attribute vectors ***x*** in TF must consist of exact *C* 1s due to one-hot encoding. Therefore, there exists only the matches *j*_1_ = *l*_1_, *j*_2_ = *l*_2_, …, *j*_*D*_ = *l*_*D*_ where all the attributes in these positions are set to 1.

#### 4.3.2. FM-Extended Models

The Factorization Machine (FM) (Rendle et al., 2011) allows numerical attributes ***x*** ∈ ℝ^*N*^ as input, including one-hot representations of users and items. Although higher-order interactions between attributes could be formulated, FM focuses on at most second-order interactions. To derive FM from (38), let 0 = *D*_*m*_ ≤ *d* ≤ *D*_*M*_ = 2 and $w_{j_{1}j_{2}}=f_{2}\left({\text{v}}_{j_{1}},{\text{v}}_{j_{2}}\right)={\text{v}}_{j_{1}}^{⊤}{\text{v}}_{j_{2}}$ in (39) be applied for the second-order interaction. We then begin to simplify (38):

(42) $$\begin{array}{l} \mu_{R}=\underset{d=0}{\underset{︸}{w_{0}}}+\underset{d=1}{\underset{︸}{\overset{N}{\underset{l=1}{\sum }}w_{l}x_{l}}}+\underset{d=2}{\underset{︸}{\overset{N}{\underset{j_{1}=1}{\sum }}\overset{N}{\underset{j_{2}=j_{1}+1}{\sum }}w_{j_{1}j_{2}}\left(x_{j_{1}}x_{j_{2}}\right)}}\\ \;=w_{0}+\overset{N}{\underset{l=1}{\sum }}w_{l}x_{l}+\overset{N}{\underset{j_{1}=1}{\sum }}\overset{N}{\underset{j_{2}=j_{1}+1}{\sum }}v_{j_{1}}^{⊤}v_{j_{2}}\left(x_{j_{1}}x_{j_{2}}\right) \end{array}$$

which is exactly the formulation of FM. Note that FM implicitly requires all the latent factor vectors ***v*** to be of the same dimension *K*; however, this requirement could be removed from the viewpoint of our general form (38). Models in this category mainly differ in two aspects. First, linear mapping can be replaced by deep neural networks, which allows non-linear mapping of attributes. Second, FM only extracts first-order and second-order interactions. Further works such as Cao et al. (2016) extract higher-order interactions between attributes.

### 4.4. Modeling User-Item-Rating Interactions Using Heterogeneous Graphs

We notice several relevant works that perform low-rank factorization or representation learning in heterogeneous graphs, such as (Lee et al., 2011; Yu et al., 2014; Pham et al., 2016; Zheng et al., 2016; Palumbo et al., 2017; Jiang et al., 2018; Nandanwar et al., 2018). Note that these works do not require graph-structured data. Instead, they model the interactions between user, item, rating, and attribute as a heterogeneous graph. The interactions of users and items can be represented by a heterogeneous graph with two node types. An edge is unweighted for implicit feedback and weighted for explicit opinions. External attributes are typically leveraged by assigning them extra nodes in the heterogeneous graph. A heterogeneous graph structure is more suitable for categorical attributes, since each candidate value of attributes can be naturally assigned a node.

In heterogeneous graphs, recommendation can be viewed as a *link prediction* problem. Predicting a future rating corresponds to forecasting whether an edge will be built between user and item nodes. The existing works commonly adopt a two-stage algorithm to learn the model. First, we apply a random-walk or a meta-path algorithm to gather the similarities between users and items from a heterogeneous graph. The similarity information can be kept as multiple similarity matrices or network embedding vectors. Then, a matrix factorization model or other supervised machine learning algorithms are applied to extract discriminative features from the gathered similarity information, which is used for future rating prediction. Another kind of method is to first define the environment where ranking or similarity algorithms are applied. Defining the environment refers to either determining the heterogeneous graph structures or learning the transition probabilities between nodes from observed heterogeneous graphs. Having the environment, we can apply an existing algorithm (Rooted PageRank, for example) or a proposed method to gain the relative ranking scores for each item. In other words, the main difference between the two kinds of methods is whether the similarity calculation is in the first stage or the second stage. Both kinds of methods as abovementioned can be unified as a constrained likelihood maximization:

(43) $$\begin{array}{l} \underset{\theta }{\text{argmax }}\underset{\text{Likelihood}}{\underset{︸}{p\left(s,R|\theta ,X\right)}}\text{such that}\\ \underset{\text{Constraint considering attributes}}{\underset{︸}{s\left(u,i\right)=\underset{w\in {\mathbb{P}}_{u,i}|X}{\sum }f_{\theta }\left(w,r_{ui}\right)\;\forall \left(u,i\right),r_{ui}\in \delta \left(R\right)}}, \end{array}$$

where a parameterized function *f*_θ_ is specifically defined to estimate a similarity score *s*(*u, i*) of item *i*, given user *u* as a query. The calculation of a similarity score comes from the set ℙ_*u,i*_ of random walks or paths *w* from node *u* to *i* in the heterogeneous graph. The generation of ℙ_*u,i*_ considers the attribute node set ***X***. Either or both of the likelihood and the constraint may involve the information of observed ratings δ(***R***) of rating matrix ***R*** for likelihood maximization or similarity calculation. In our estimation, the current heterogeneous-graph-based models do not directly solve the constrained optimization problem (Equation 43). Commonly, they first exploit a two-stage solution that either solves the likelihood maximization or satisfies the similarity constraint. The output is then cast into the other part of (Equation 43). With different definitions of *f*_θ_ and *p*, the two-stage process may run only once or iteratively until convergence. The definition of *s*(*u, i*) in the surveyed papers includes PageRank (Lee et al., 2011; Jiang et al., 2018), PathSim (Yu et al., 2014), and so on. The likelihood function *p* guides the similarity-related parameters θ to fit the distribution objective of observed similarities *s* or ratings ***R***. The objective may be given attributes ***X*** as auxiliary learning data. Minor works like (Lee et al., 2011) do not optimize the likelihood; instead, they directly compute the similarity constraint with pre-defined θ from a specifically designed heterogeneous graph.

We now explain why random walk or path-based algorithms in heterogeneous graphs are regarded as collaborative filtering methods. For ease of explanation, we first consider the case of no auxiliary attributes. We have users and items as nodes in a graph structure, where edge weights denote the ratings of users toward items. If both users *u* and *v* rate the same item *i*, then *i* becomes a shortcut from *u* to *v*. Therefore, starting from user node *u*, another user *v* at a low shortest path distance from *u* could have similar rating behaviors as *u*. We can then recommend items at short distances from *u*, based on the shortcut through *v*. This is just the spirit of collaborative filtering, which exploits the similar rating behaviors of other users for future recommendation to target users. If attribute nodes are taken into consideration in heterogeneous graphs, they also become the shortcuts for paths between users and items.

- **HeteRec** (Yu et al., 2014). This model first assumes an attribute-aware heterogeneous graph formed by attributes and ratings. We then obtain *M* non-negative PathSim Sun et al. (2011) similarity matrices ***S***^(1)^, ***S***^(2)^, …, ***S***^(*m*)^, …, ***S***^(*M*)^. Given low-rank non-negative factorization of each ***S***^(*m*)^ = ***U***^(*m*)⊤^***V***^(*m*)^, a rating estimate $\hat{r}$ is defined as follows:
  (44) $$\begin{array}{l} \hat{r}=\overset{M}{\underset{m=1}{\sum }}\theta_{m}u^{\left(m\right)⊤}v^{\left(m\right)}. \end{array}$$
- **Graph-Based Flexible Recommendation (GFREC)** (Lee et al., 2011). This approach applies personalized PageRank, an unsupervised random walk-based algorithm, to perform random walks in a bipartite heterogeneous graph for recommendation. Instead of independently defining a single node for each categorical attribute value, GFREC makes a node imply both an attribute value and its associated user or item. For example, given a user *u* and its corresponding attribute value *x*, we can put a node named (*u, x*) in the heterogeneous network. In a GFREC bipartite heterogeneous graph, two disjoint sets respectively refer to users and items. GFREC shows that personalized PageRank can compute the visiting probabilities of each node in this bipartite heterogeneous graph. Finally, the probabilities are used to rank items to be recommended.

### 4.5. Differences Between Models

In our classification system, there are still a number of works in each category. Although models in the same category share a similar mathematical form in terms of the design of the objective function, they can vary in certain design aspects. One of the most important differences is the task they focus on. Some models emphasize predicting future ratings. Therefore, they are usually dedicated to minimizing the *Root Mean Square Error (RMSE)* to achieve a more accurate prediction of scores. Some other models care more about the top-N items that a user may like. Hence, they adopt pairwise ranking to predict the preference for items of a given user. A second difference is based on the types of attributes that are exploited. For example, Yang et al. (2011) takes a social network as its input feature matrix. A third difference is the source of attributes each model claims to use. Some models claim to accept only user attributes, while others might be more general for different types of attributes.

## 5. Empirical Comparison

In this section, we evaluate the effectiveness of each model by examining its performance on several datasets. We focus on the *rating prediction* task since the majority of the models have their objectives designed for this task. We also compare the performance of each competitor under different conditions: with/without user-relevant attributes, item-relevant attributes, or rating-relevant attributes. Hyperparameters for each model are tuned based on grid search.

### 5.1. Experimental Setup

#### 5.1.1. Model

We consider several popular models that are representative of each category for comparison: the Regression-based Latent Factor Model (RLFM) (Agarwal and Chen, 2009) and Friendship-Interest Propagation (FIP) (Yang et al., 2011) in Discriminative Matrix Factorization, Collective Matrix Factorization (CMF) (Singh and Gordon, 2008) in Generative Matrix Factorization, and Tensor Factorization (TF) (Karatzoglou et al., 2010), the Factorization Machine (FM) (Rendle et al., 2011), and the Neural Factorization Machine (NFM) (He and Chua, 2017) in Generalized Factorization. We also select Matrix Factorization (MF) (Chin et al., 2016) as a simple baseline model that does not include any attribute and Neural Collaborative Filtering (NCF) (He et al., 2017) as a stronger baseline (the simple version where attributes are one-hot encoding vectors of users and items). NCF+ (where attributes are one-hot encoding vectors appended with those from datasets) serves as a competitor in DMF. We do not compare Heterogeneous Graph models, as models in this category are more diverse and it is hard to pick a representative model. The attribute types that each model accepts are summarized in Table 6.

**Figure 17:**
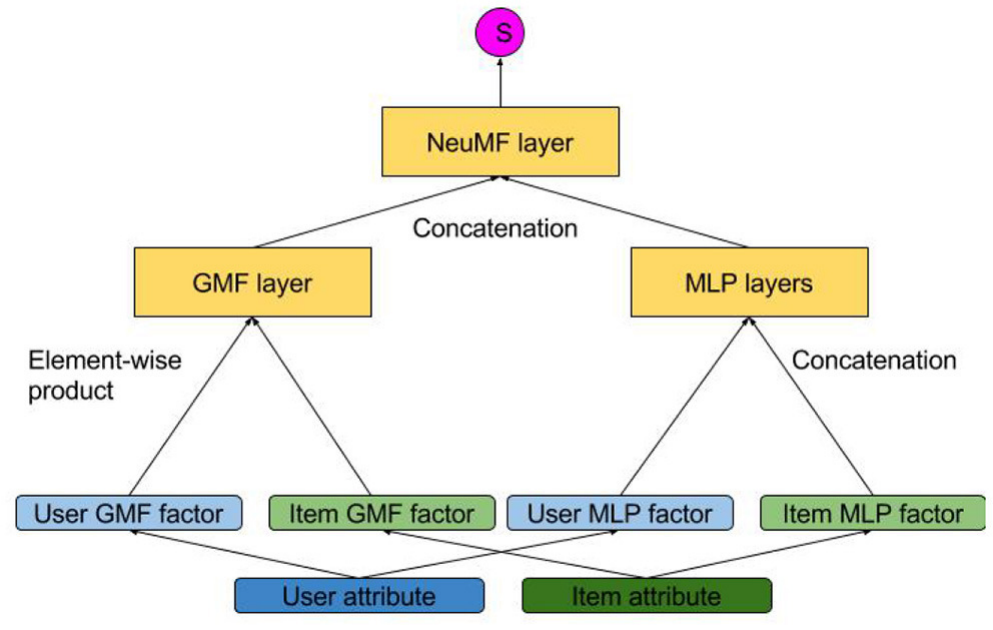
Model structure of NCF.

- **Tensor Factorization (TF)**
  TF is an *D*-dimensional extension of MF. We denote the tensor containing the ratings by $R\in {\mathbb{R}}^{N_{1}\times N_{2}\times \ldots \times N_{D}}$. The tensor R can be factorized into *D* matrices ${\text{V}}_{j}\in {\mathbb{R}}^{K_{j}\times N_{j}}$ and one central tensor $S\in {\mathbb{R}}^{K_{1}\times K_{2}\times \ldots \times K_{D}}$ where *K*_1_, *K*_2_, …, *K*_*D*_ is the dimension of latent factors. In this case, the predicted rating for *r*_*j*_1_*j*_2_…*j*_*D*__ is ${\hat{r}}_{j_{1}j_{2}\ldots j_{D}}=S\times_{V_{1}}V_{1}\times_{V_{2}}V_{2}\times \ldots \times_{V_{D}}V_{D}$. Note that the subscript of the tensor-matrix multiplication operator × _***V***_ shows the direction on which the tensor multiplies the matrix. The loss function for this model is
  (45) $$\begin{array}{l} \underset{S,V}{\text{argmin}}\underset{j_{1},j_{2},\ldots ,j_{D}|r_{j_{1}j_{2}\ldots j_{D}}\in \delta \left(R\right)}{\sum }{\left({\hat{r}}_{j_{1}j_{2}\ldots j_{D}}-r_{j_{1}j_{2}\ldots j_{D}}\right)}^{2}\\ +\overset{D}{\underset{j=1}{\sum }}\Omega \left(V_{j}\right)+\Omega \left(S\right), \end{array}$$
  where δ(R) is the set of non-missing entries in R, and $\Omega \left(\text{V}\right)=\frac{\lambda_{V}}{2}∥\text{V }{∥}_{F}^{2}$ is the regularization term of squared Frobenius norm. We can update the latent factors using SGD. One major concern of with model is that its complexity and storage requirement grow exponentially with the number of dimensions of the rating tensor R.
- **Collective Matrix Factorization (CMF)**
  CMF is a model incorporating side information by factorizing multiple matrices simultaneously. In an *D*-entities schema, ${\text{X}}^{\left(ij\right)}\in {\mathbb{R}}^{N_{i}\times N_{j}}$ represents the relation between entity *i* and *j* if the relation exists, i.e., *E*_*i*_ ~ *E*_*j*_. CMF factorizes these matrices into ${\text{U}}^{\left(1\right)}\in {\mathbb{R}}^{K\times N_{1}},{\text{U}}^{\left(2\right)},\ldots ,{\text{U}}^{\left(D\right)}\in {\mathbb{R}}^{K\times N_{D}}$ such that ***X***^(*ij*)^ ≈ *f*^(*ij*)^(***U***^(*i*)⊤^***U***^(*j*)^). For a dataset with user- and item-relevant attributes, there are four entities (*E*_1_: user id, *E*_2_: item id, *E*_3_: user features, and *E*_4_: item features) and three relations (***X***^(12)^: ratings matrix, ***X***^(13)^, ***X***^(24)^: feature matrix). In our experiment, f is the identity function for the rating matrix and the sigmoid function is that for the feature matrix. Let *E* = {(*i, j*):*E*_*i*_ ~ *E*_*j*_ ∩ *i* < *j*} denote the set of all existing relations pairs, ***U*** denote the set of latent factors, ***W*** denote the set of weight matrices, and $D_{F}\left(\text{Y}||\text{X},\text{W}\right)=\underset{ij}{\sum }w_{ij}\left(F\left(y_{ij}\right)+F^{*}\left(x_{ij}\right)-y_{ij}x_{ij}\right)$ measure the weighted divergence of two matrices ***Y*** and ***X***. The loss function for this model is
  (46) $$\begin{array}{l} \underset{U,W}{\text{argmin}}\sum_{ij\in E}\alpha^{\left(ij\right)}(D_{F^{\left(ij\right)}}(U^{(i)⊤}U^{\left(j\right)})||X^{\left(ij\right)},W^{\left(ij\right)})\\ +D_{G^{\left(i\right)}}(0||U^{\left(i\right)})+D_{G^{\left(j\right)}}(0||U^{\left(j\right)})) \end{array}$$
  where *F*^(*ij*)^ defines the loss for a reconstruction, and *G*^(*i*)^ defines the loss for a regularizer. We can update ***U*** through a Newton-Raphson step.
- **Regression-Based Latent Factor Model (RLFM)**
  Let *r*_*ui*_ denote the rating given by user *u* to item *i*. ${\text{z}}_{\pi \left(u,i\right)}\in {\mathbb{R}}^{K_{Z}}$, ${\text{x}}_{u}\in {\mathbb{R}}^{K_{X}}$ and ${\text{y}}_{i}\in {\mathbb{R}}^{K_{Y}}$ denote attribute vectors for rating π(*u, i*) (i.e., index associated with user *u* and item *i*), user *u*, and item *i*, respectively. This model learns the latent factors ($\alpha_{u}\in \mathbb{R},{\text{w}}_{u}\in {\mathbb{R}}^{K}$) to user *u*, ($\beta_{i}\in \mathbb{R},{\text{h}}_{i}\in {\mathbb{R}}^{K}$) to item *i* and ($\text{b}\in {\mathbb{R}}^{K_{Z}}$) to rating *r*_*ij*_, such that the rating is estimated by:
  (47) $$\begin{array}{l} {\hat{r}}_{ij}=z_{\pi \left(i,j\right)}^{⊤}b+\alpha_{u}+\beta_{i}+w_{u}^{⊤}h_{i} \end{array}$$
  This model assumes that α_*u*_, β_*i*_, ***w***_*u*_, and ***h***_*i*_ follow Gaussian distribution given attributes ***x***_*u*_ and ***y***_*i*_, so the model can be fitted by a Monte Carlo EM algorithm.
- **Friendship-Interest Propagation (FIP)**
  FIP combines learned latent factors (***W***, ***H***) and a given attribute matrix (***X***, ***Y***) to fit user profiles and item properties. Let *U* be the set of users and *I* be the set of items. For each training example (*u, i, r*) ∈ *O*, user *u* ∈ *U* gives item *i* ∈ *I* a rating *r*. The objective function is as follows:
  (48) $$\begin{array}{l} \underset{W,H,C}{\text{argmin}}\underset{\left(u,i,r\right)\in O}{\sum }L(r,w_{u}^{⊤}h_{i}+x_{u}^{T}Cy_{i})+{\text{λ}}_{C}\Omega (C)\\ +{\text{λ}}_{W}(\Omega (W)+\Omega (w_{u}-Ax_{u}))+{\text{λ}}_{H}(\Omega (H)\\ +\Omega (h_{i}-By_{i}))+{\text{λ}}_{A}\Omega (A)+{\text{λ}}_{B}\Omega (B) \end{array}$$
  where $L\left(r,\hat{r}\right)$ is a loss function, ***C*** is a correlation matrix, ***A*** and **B** are the correlation matrices between attribute and latent factors, Ω(·) is a regularization term, and all of the λ with subscripts are hyperparameters. If both user and item attributes are not given, the model is then reduced to matrix factorization. Since it is often the case that a dataset contains either user or item attributes, in the experiments, if a user (or item) attribute is not given, we assume it is a vector of ones with the same dimension as item (or user).
- **Factorization Machine (FM)**
  FM reduces the original recommendation problem into a traditional classification (or regression) problem. For example, for each observation (*u, i, r*) ∈ *O*, it can be transformed into an attribute vector ***x*** (which can be formed by representing user *u* and item *i* as two one-hot encoding vectors and concatenating them together) and a target rating *r*. The goal is then to fit the target value by utilizing the attribute vector. The objective function can be addressed as follows:
  (49) $$\begin{array}{l} \underset{w,V}{\text{argmin}}\underset{\left(u,i,r\right)\in O}{\sum }L\left(r,w_{0}+\overset{N}{\underset{i=1}{\sum }}w_{i}x_{i}+\overset{N}{\underset{i=1}{\sum }}\overset{D}{\underset{j=i+1}{\sum }}\left(\overset{K}{\underset{k=1}{\sum }}v_{ik}v_{jk}\right)x_{i}x_{j}\right)\\ +\lambda_{w}\Omega \left(w\right)+\lambda_{V}\Omega \left(V\right) \end{array}$$
  where ***w*** is the weight vector (*w*_*i*_ is its *i*-th element) and ***V*** ∈ ℝ^*K × N*^ is the latent factor matrix. This is called a *factorization machine of degree 2* (or *two-way factorization machine*). An *N-way factorization machine* can be expressed as follows:
  (50) $$\begin{array}{l} \underset{w,V}{\text{argmin}}\underset{\left(u,i,r\right)\in O}{\sum }L(r,w_{0}+\overset{N}{\underset{i=1}{\sum }}w_{i}x_{i}\\ +\overset{N}{\underset{l=2}{\sum }}\overset{N}{\underset{i_{1}=1}{\sum }}\overset{N}{\underset{i_{2}=i_{1}+1}{\sum }}\cdots \overset{N}{\underset{i_{l}=i_{l-1}+1}{\sum }}(\overset{K}{\underset{k=1}{\sum }}\overset{l}{\underset{j=1}{\prod }}v_{i_{j}k})\overset{l}{\underset{j=1}{\prod }}x_{i_{j}})\\ +\lambda_{w}\Omega \left(w\right)+\lambda_{V}\Omega \left(V\right). \end{array}$$
  In our experiments, only a two-way factorization machine is used as our baseline model, since it is the most frequent configuration in the experiments conducted in previous studies.
- **Neural Collaborative Filtering (NCF)**
  NCF (Figure 17) consists of two parts: generalized matrix factorization (GMF) and multi-layer perceptron (MLP). The GMF layer computes the element-wise product of user and item latent factors. The MLP layer is a neural network that takes the concatenation of user and item latent factors as inputs and outputs a vector. The results of GMF and MLP are then concatenated as a vector and serve as the input of the NeuMF layer, which is a one-layer perceptron and outputs the predicted rating. Normally, a user/item attribute is a one-hot encoding vector that represents the user/item. However, if external attributes are provided, they can be easily modified.
- **Neural Factorization Machine (NFM)**
  NFM is a generalization of two-way FM. While FM extracts the linear interaction between attributes, NFM is able to extract non-linear interactions with the help of a non-linear activation function in a deep neural network. The objective of NFM can be expressed as follows:
  (51) $$\begin{array}{l} \underset{w,V,f}{\text{argmin}}\underset{\left(u,i,r\right)\in O}{\sum }L\left(r,w_{0}+\overset{N}{\underset{i=1}{\sum }}w_{i}x_{i}+f\left(\overset{N}{\underset{i=1}{\sum }}\overset{N}{\underset{j=i+1}{\sum }}x_{i}v_{i}⊙x_{j}v_{j}\right)\right)\\ +\lambda_{w}\Omega \left(w\right)+\lambda_{V}\Omega \left(V\right)+\lambda_{f}\Omega \left(f\right) \end{array}$$
  where ⊙ is element-wise product of vectors and ***f*** is the neural network. The neural network takes second-order interactions of attribute vectors in FM as input. In fact, FM can be reduced from NFM where ***f*** is a vector of ones.

**Table 6:** Attribute types that are used for each model.

**Model**	**U**	**I**	***R***
TF	✓	✓	✓
CMF	✓	✓	
RLFM	✓	✓	✓
FIP	✓	✓	
FM	✓	✓	✓
NCF	✓	✓	
NFM	✓	✓	✓
MF			

*U, user-relevant attribute; I, item-relevant attribute; R, rating-relevant attribute*.

#### 5.1.2. Dataset

We use widely used data available online to compare the performance of the models. Here, we briefly introduce these datasets, and their statistics can be seen in Tables 7–9. For each dataset, we use the original training and testing sets if they are provided by the host. Otherwise, the training set and testing sets are split based on timestamps, where the training set represents past data and the testing set represents future ratings.

- **MovieLens-1M, 10M, 20M** (Harper and Konstan, 2015)
  The MovieLens datasets contain ratings that users give to different movies with different numbers of ratings (i.e., 1, 10, and 20M). They also include certain user information, such as genre, age and occupation, and item information, for example, the genre of a movie and the year when the movie was produced. The training and testing sets are divided by the time that the ratings were generated. The latest-timestamped 10% ratings serve as the testing set, while the others are served as the training set.
- **Netflix**^1^
  The Netflix Prize is a competition dedicating to the design of a better movie recommendation system. The data that the host provides contain a large amount of rating instances. It also includes side information about the movies. The testing set is extracted from the probe set, which the host has provided. However, since the training set is so large that several of the models that we are testing cannot finish training in a reasonable amount of time, it is randomly sampled to one-tenth of the original size in all of our experiments.
- **Yahoo Music**^2^
  Yahoo provides two music datasets (denoted by Yahoo Music 1 and 2 in our experiments) for researchers to study how users rate music products, including tracks and albums. Information such as the genre or artist of a product is provided. The data were also used in the KDD Cup 2011. Among the items being rated in the original competition (albums, tracks), we extract tracks as targets to be rated. The training and testing sets are split in the way provided by the host.
- **Yelp**^3^
  The Yelp Challenge is a contest that allows participants to come up with a research topic themselves based on the given Yelp dataset. The dataset contains ratings given by users to businesses. It includes user information and item information of various types. The reviews that users give to items are also included. The training and testing sets are split in the same way as we did in the MovieLens datasets.

**Table 7:** Basic statistics of datasets.

**Dataset**	**Users**	**Items**	**Training ratings**	**Test ratings**	**Density**
MovieLens-1M	6040	3883	900188	100021	3.84 × 10^−2^
MovieLens-10M	69878	10681	9000048	1000006	1.21 × 10^−2^
MovieLens-20M	138493	10378	17819935	1979993	1.24 × 10^−2^
Netflix	475708	17770	9907271	1408394	1.17 × 10^−3^
Yahoo Music 1	129100	4772	702947	6858	1.14 × 10^−3^
Yahoo Music 2	50751	3852	367556	7249	1.88 × 10^−3^
Yelp	1029432	135086	3635310	406952	2.61 × 10^−5^

*We define $\text{Density}\;as\frac{\text{\#(training ratings)}}{\text{\#(users)}\times \text{\#(items)}}$*.

**Table 8:** Attribute statistics of datasets.

**Dataset**	***U***	**I**	***R***
MovieLens-1M	29	99	0
MovieLens-10M	0	112	0
MovieLens-20M	0	220	0
Netflix	0	95	0
Yahoo Music 1	0	300	0
Yahoo Music 2	0	300	0
Yelp	18	234	3

*0 means the type of attribute is not present in this dataset*.

**Table 9:** Percentage of new users/items (users/items in testing data but not in training data).

**Dataset**	**% of new users**	**% of new items**
MovieLens-1M	2.4	0.8
MovieLens-10M	65.5	10.8
MovieLens-20M	73.1	8.5
Netflix	4.8	0
Yahoo music 1	61.1	0
Yahoo music 2	46.3	0
Yelp	49.8	3.0

#### 5.1.3. Attribute Extraction

Most models accept real value attributes as their input. For categorical attributes, the value merely represents which category the user/item belongs to, which means that there is no physical meaning to the value. Therefore, each category is treated as a new attribute dimension. For each dimension, if the user (or item) is in this category, the value is 1, and otherwise 0 (i.e., one-hot encoding). However, categorical attributes are not used in the TF model due to its high space complexity. Since one-hot encoding significantly increases the dimensions of attributes, we find that many of the experimented models cannot finish training in a reasonable amount of time for large-scale datasets. Hence, we determine to retain only the top 100 representative transformed attributes that have the most values of 1. For the Yelp dataset, since some of its attribute values vary significantly, log(1 + *x*) is applied if the original attribute value x is positive, and −log(−*x*) is applied for negative ones. For TF, the attribute value is further rounded to the nearest integer. For the MovieLens-1M dataset, we combine user attributes with item attributes. For the Yelp dataset, we separate user, item, and rating attributes and compare the results of different combinations of these attributes. For all the other datasets, we use item attributes only.

#### 5.1.4. Evaluation Metric

*Root Mean Square Error (RMSE)* [defined in (19)] is selected as the evaluation metric in our experiments. RMSE is arguably the most widely used evaluation metric for rating prediction, since most model-based collaborative filtering methods try to minimize MSE (RMSE without root) or RMSE as their objectives.

### 5.2. Performance Comparisons

We run seven benchmark models on seven attribute-appended rating datasets. All the empirical comparisons, evaluated with RMSE, are reported in Tables 10, 11. Note that the bold values are best performances in that caetgory among different models and the star symbol (*) indicates excessive running time or high memory usage (over 24 h or more than 64 GB memory); this usually happens when TF runs on data with a large number of features. MF and NCF are trained on ratings only. We can see that RLFM, FM, NFM, and NCF+ can outperform the basic MF models on all datasets. In general, NCF+ yields the best results among these models.

**Table 10:** RMSE on all datasets except Yelp.

**Dataset**	**MF**	**NCF**	**TF**	**CMF**	**RLFM**	**FIP**	**FM**	**NFM**	**NCF+**
ML-1M	0.9002	0.9082	*	0.9088	0.8824	0.9396	**0.8798**	0.9162	0.9054
ML-10M	0.9820	0.9161	*	0.9763	0.9111	1.1085	**0.9103**	0.9132	0.9129
ML-20M	0.9923	0.9402	*	0.9954	**0.9227**	1.1128	0.9297	0.9260	0.9240
Netflix	1.2033	1.0737	1.1434	1.0848	1.1325	1.1312	1.0887	1.0707	**1.0705**
Yahoo 1	34.9989	33.0522	*	34.3325	**32.9302**	35.8085	33.1422	33.9271	33.1743
Yahoo 2	46.8444	41.2785	*	45.2139	45.3166	50.6670	45.4528	42.2594	**40.4920**

**Table 11:** RMSE on Yelp.

**Baselines**	**Attribute**	**TF**	**CMF**	**RLFM**	**FIP**	**FM**	**NFM**	**NCF+**
MF: 1.4809 NCF:1.3805	U	*	1.3967	1.1434	1.4162	1.1337	1.1440	**1.1280**
	I	*	1.3951	1.2672	1.4269	1.2849	1.2923	**1.2586**
	U+I	*	1.3848	1.1029	1.2905	1.0603	1.0876	**1.0372**
	R	1.4958	–	1.3114	–	**1.3065**	1.3386	–
	U+R	*	–	1.1244	–	**1.1067**	1.1168	–
	I+R	*	–	**1.2470**	–	1.2566	1.2693	–
	U+I+R	*	–	1.0852	–	**1.0372**	1.0755	–

### 5.3. Findings and Discussion

In this section, we give a brief comparison in Table 12 and discuss some findings from the experiment results in section 5.2.

- Attribute-aware models outperform their counterparts that do not incorporate attributesWe first identify two pairs of recommender systems: MF with FM and NCF with NCF+. FM can be seen as a design that incorporates attributes into MF, and the same applies for NCF+ vs. NCF. From Tables 10, 11, we can see that in most cases, attribute-aware designs (FM, NCF+) do outperform their counterparts (MF, NCF). This is intuitive, as attributes can provide additional information that benefits recommendation performance. Therefore, for each of the two pairs, attribute-aware design can achieve better performance.
- DMF and GF models perform better than GMF in general The results in Tables 10, 11 show that discriminative matrix factorization models (TF, RLFM, and NCF+) and matrix factorization generalization (FM and NFM) perform better than a generative matrix factorization design (CMF). This may be because, in addition to reconstructing a rating matrix, generative matrix factorization models have to simultaneously recover attribute matrices, which could lead to overfitting when given insufficient data.
- User attributes are more beneficial than item and rating attributesAs the dataset contains three kinds of attributes (user, item, and rating), we would focus our discussion based on the result of Table 11. RLFM, FM, and NFM are the only models that consider all three types of attributes, and it is apparent that the best result occurs when all attributes are exploited. However, if we consider three types of attribute separately, it seems that the user attributes are the most beneficial to most models. The result is expected as the goal of recommender systems is to predict user preferences regarding items. Therefore, user information should play the most important role. Rating attributes are not as effective as the others, and NCF+ can achieve top performance without using them.

**Table 12:** Comparison between three types of models.

**Models**	**DMF**	**GMF**	**GF**
General attributes	No	No	Yes
Predicting missing attributes	No	Yes	No
Performance	Good	Not as Good	Good

## 6. Conclusion

Collaborative filtering is arguably the most effective approach to building a recommender system. This paper introduces and compares the performance of different attribute-aware models. We believe it can benefit not only researchers in this area but also engineers intending to build a recommender system that utilizes a rich set of attributes.

## Author Contributions

All authors listed have made a substantial, direct and intellectual contribution to the work, and approved it for publication.

### Conflict of Interest

The authors declare that the research was conducted in the absence of any commercial or financial relationships that could be construed as a potential conflict of interest.

## Footnotes

^1^http://www.netflixprize.com/

^2^https://webscope.sandbox.yahoo.com/

^3^https://www.yelp.com/dataset
